# Artificial Intelligence (AI) in Breast Imaging: A Scientometric Umbrella Review

**DOI:** 10.3390/diagnostics12123111

**Published:** 2022-12-09

**Authors:** Xiao Jian Tan, Wai Loon Cheor, Li Li Lim, Khairul Shakir Ab Rahman, Ikmal Hisyam Bakrin

**Affiliations:** 1 Centre for Multimodal Signal Processing, Tunku Abdul Rahman University of Management and Technology (TAR UMT), Jalan Genting Kelang, Setapak, Kuala Lumpur 53300, Malaysia; 2 Department of Electrical and Electronics Engineering, Faculty of Engineering and Technology, Tunku Abdul Rahman University of Management and Technology (TAR UMT), Jalan Genting Kelang, Setapak, Kuala Lumpur 53300, Malaysia; 3 Sports Engineering Research Centre (SERC), Universiti Malaysia Perlis (UniMAP), Arau 02600, Malaysia; 4 TE Connectivity Operations Sdn. Bhd, Perai 13600, Malaysia; 5 Faculty of Electronic Engineering Technology, Universiti Malaysia Perlis (UniMAP), Arau 02600, Malaysia; 6 Department of Pathology, Hospital Tuanku Fauziah, Jalan Tun Abdul Razak, Kangar 01000, Malaysia; 7 Department of Pathology, Faculty of Medicine and Health Sciences, Universiti Putra Malaysia, UPM Serdang, Serdang 43400, Malaysia

**Keywords:** artificial intelligence, deep learning, machine learning, breast imaging, mammogram, scientometric analysis, umbrella review

## Abstract

Artificial intelligence (AI), a rousing advancement disrupting a wide spectrum of applications with remarkable betterment, has continued to gain momentum over the past decades. Within breast imaging, AI, especially machine learning and deep learning, honed with unlimited cross-data/case referencing, has found great utility encompassing four facets: screening and detection, diagnosis, disease monitoring, and data management as a whole. Over the years, breast cancer has been the apex of the cancer cumulative risk ranking for women across the six continents, existing in variegated forms and offering a complicated context in medical decisions. Realizing the ever-increasing demand for quality healthcare, contemporary AI has been envisioned to make great strides in clinical data management and perception, with the capability to detect indeterminate significance, predict prognostication, and correlate available data into a meaningful clinical endpoint. Here, the authors captured the review works over the past decades, focusing on AI in breast imaging, and systematized the included works into one usable document, which is termed an umbrella review. The present study aims to provide a panoramic view of how AI is poised to enhance breast imaging procedures. Evidence-based scientometric analysis was performed in accordance with the Preferred Reporting Items for Systematic reviews and Meta-Analyses (PRISMA) guideline, resulting in 71 included review works. This study aims to synthesize, collate, and correlate the included review works, thereby identifying the patterns, trends, quality, and types of the included works, captured by the structured search strategy. The present study is intended to serve as a “one-stop center” synthesis and provide a holistic bird’s eye view to readers, ranging from newcomers to existing researchers and relevant stakeholders, on the topic of interest.

## 1. Introduction

**Figure 1:**
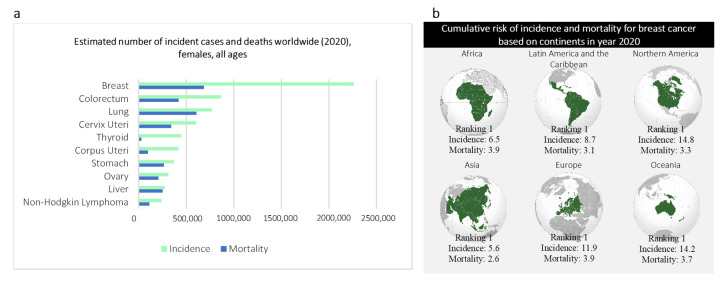
Breast cancer statistics as of 2020 [3]. (**a**), estimated number of incidence cases and deaths worldwide in year 2020 (females, all ages). (**b**), Cumulative risk of incidence and mortality for breast cancer based on continents in year 2020.

Breast cancer, the first and oldest description of cancer found in history, was discovered in Egypt in approximately 3000 BC, where the term “cancer” remains in use [1,2]. The ancient Egyptian textbook on trauma surgery describes eight cases of ulcers or tumors cauterized using a fire drill with the conclusion: there is no treatment [1,2]. To date, breast cancer remains a global challenge, proclaimed as the leading mortality (cancer) with 684,996 deaths (15.5% of all cancer cases amongst women) reported across the world in 2020 [3]. Breast cancer is the most frequently diagnosed cancer among women, regardless of socioeconomic, geographic subgroups, race, and ethnicity [3,4] (Figure 1). Breast cancer is a non-communicable disease that emerges in variegated forms, self-subsistent, and interacts dynamically through an adaptive process with its microenvironment. Given this complexity, breast imaging modalities have become a pivotal component for every stage in cancer management, starting from the initial cancer detection, followed by accurate demarcation of neoplastic lesions, treatment response assessment, and post-treatment follow-up. The introduction of promising imaging modalities (e.g., mammograms) has reshaped the landscape and horizon of cancer management. Early detection of breast cancer using mammograms in conjunction with advanced treatment have been proven to decrease mortality by 30.0% as of 1989 [5,6]. This finding is supported by recent incident-based mortality and randomized trial studies finding women with mammogram screening have a statistically significant reduction in mortality and probability of developing advanced breast cancer by 41.0% and 25.0%, respectively [7].

When a suspicious lesion is detected by an imaging modality, a series of cascading clinical procedures of observation, testing, and/or empiric biopsy is initiated. According to the standard clinical protocol, the suspicious lesions would be demarcated, distinguished from the non-neoplastic lesion/mimicker, and classified into different cancer aggressiveness levels. The optimal treatment is then proposed while a temporal monitor of the cancer is performed from time to time. Although there are several exceptions (e.g., imaging using nuclear medicine whereby quantitative measurement (i.e., metabolic activity) can be obtained based on specific uptake value), the conventional breast imaging evaluation relies mainly upon the qualitative features (e.g., shape, size, and density of cancer). These features are collectively termed semantic features [8,9]. Thanks to the advancement in computational mathematics, now, the semantic features can be modeled and quantified, collectively forming the core tenets of radiomics [10,11,12]. Radiomics, also known as quantitative imaging [13,14,15,16], is equipped with the high-throughput extraction of quantitative data, which empowers the conversion of qualitative image features into mineable data [17,18,19]. As a voluminous amount of multi-dimensional data is made available via this advancement, the efficiency and efficacy of data interpretation may pose a challenge to medical experts. To fully utilize the extracted data as a meaningful clinical endpoint, AI could make substantial strides in data management and data perception.

AI methodologies, particularly machine learning and deep learning, have demonstrated remarkable improvements across multiple spectrums of application. Recently, numerous studies have highlighted the superiority of deep learning (a subset of machine learning) against ground truth labels and, in some task-specific cases, surpassing the conventional expert-oriented methods [20,21,22,23,24,25]. Despite the data-hungry nature of deep learning, it offers comprehensive management and correlation of concentrated multivariate data, which potentially have no limit in cross-data/case referencing. Owing to this, the concentrated semantic data mineable from radiomics which essentially describes the radiographic aspects of the breast would be an ideal scientific-rich resource for training a deep learning model. Furthermore, deep learning allows better generalizability characteristics across different imaging modalities, high robustness to noise, increases efficiency, reduces the error rate, as well as enables and enriches data visualization.

At the dawn of this rousing technological advancement, this article aims to provide a panoramic view of how AI is positioned to aid in breast imaging procedures. Over the years, increasing interest in AI integration within breast imaging has captured the attention of numerous researchers in surveying and reviewing such integration along different facets. To avoid reinventing the wheel, the authors intended to connect these review works and focus on collating and synthesizing new insights and drawing constructive conclusions while highlighting the research gaps, challenges, and potential future direction. The idea of investigating review work as the research object is unique. To systematize this purpose into one usable document, an umbrella review [26,27] was adopted and aided with scientometric analysis [28] for comprehensive qualitative and quantitative synthesis activities purposes. This study aims to provide a brief yet broad overview alongside a holistic bird’s eye view to the readers, ranging from newcomers to existing researchers and relevant stakeholders, on the topic of interest.

### 1.1. Breast Imaging Modalities

Back in 1913, Albert Salomon, one of the pioneers of mammograms, a surgeon as well as emeritus professor had reported his impactful monograph on the radiographic investigation of mastectomy specimens. Albert Salomon’s work reveals that highly infiltrating cancer was distinguishable from circumscribed cancer [29]. These remarkable efforts were recommenced by Otto Kleinschmidt in 1927. Subsequently, in late 1960, a self-contained mobile mammographic unit was first developed by Philip Strax [30]. This mammographic unit was capable of providing mammography services to 70 women per day. To date, mammograms remain the mainstay in breast imaging [9,31,32,33].

**Figure 2:**
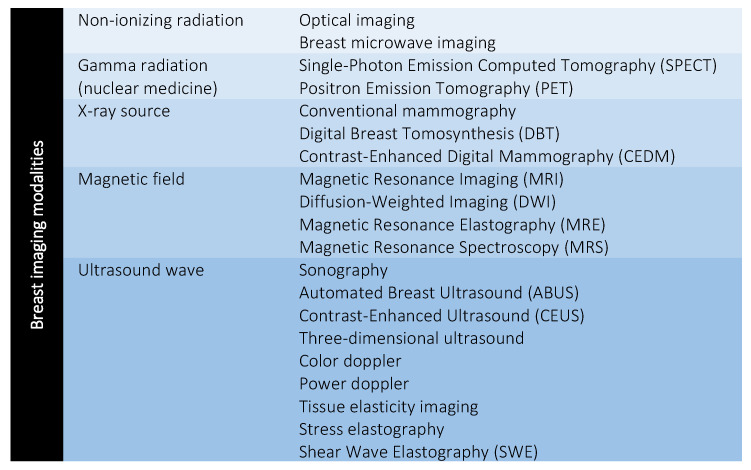
Breast imaging modalities. Figure reproduced from [34] under Creative Commons CC BY license, Springer Nature.

Over the years of ever-increasing demand for quality healthcare, researchers have actively sought promising imaging modalities as adjunctive imaging modalities to complement screening outputs. The mammogram is reported with a sensitivity of 68.0%–90.0% and approximately 62.0% for women at the age of 50 and 40–49, respectively [34]. The sensitivity of mammography is found to decrease to 57.0% for women with dense breasts but increased up to 93.0% for women with breasts in high adipose tissue [35]. In 2000, the first digital mammogram, namely Senographe 2000 D was approved by the Food and Drug Administration (FDA). Senographe 2000 D is equipped with an X-ray generator where the image acquisition method is similar to the conventional film-screen mammogram [29]. In later years, the digital mammogram has advanced to novel digital breast tomosynthesis (DBT) and contrast-enhanced digital mammography (CEDM). Other promising propitious imaging modalities (e.g., ultrasonography and magnetic resonance imaging (MRI)) are gradually introduced to use as adjuncts to mammograms for breast cancer screening. Several randomized trial studies have highlighted the applicability of these adjunctive modalities in complementing the sensitivity of mammograms, especially in the detection of early-stage and infiltrating cancers for asymptomatic women, regardless of breast density [36,37,38]. To date, within breast cancer, there are approximately 20 imaging modalities available to improve screening sensitivity and support medical experts with assistive information. Figure 2 summarizes the breast imaging modalities, clustered in accordance with different sources of radiation.

### 1.2. AI in Breast Imaging

The idea of critical thinking and mimicking intelligence behavior was first conceived by Alan Turing back in 1950 [39]. The phrase “artificial intelligence” was later coined by John McCarthy in 1956 to specifically denote the subject area of science and engineering in machine intelligence [39,40,41]. Over the years, continuous exploration in AI has concretely reshaped, evolved, and broadly encompassed applications in everyday life. Within breast imaging, advancements in both computers and various imaging modalities have synergically led to rapid progress in AI integration.

**Figure 3:**
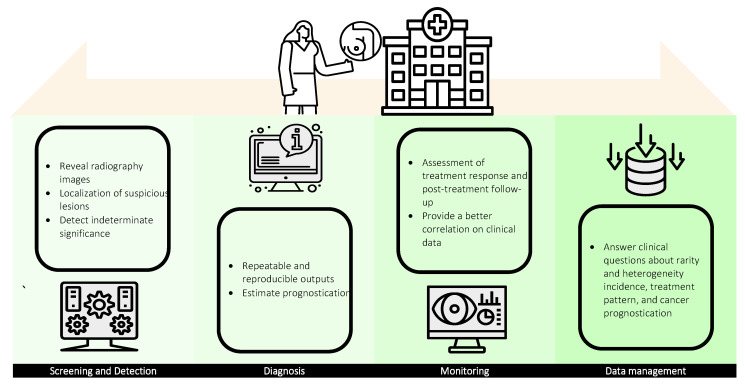
Utility of AI in breast imaging: screening and detection, diagnosis, monitoring, and data management.

AI finds great value in cancer management, comprising four facets: screening and detection, diagnosis, monitoring, and data management as a whole (Figure 3). Screening is a clinical procedure to reveal radiography images of the breast using breast imaging modalities while detection refers to the localization of objects of interest (e.g., signs of cancer) in these images. Both screening and detection can be characterized under the umbrella term of computer-aided detection (CADe). AI-assisted screening methodologies have been found to improve radiography image quality and facilitate subsequent detection and diagnosis procedures in suspicious lesions [42,43]. Although AI-based detection tools are no more accurate than radiologists in cancer detection [44,45], CADe offers consistent sensitivity in the detection of subtle changes, especially in indeterminate significance [46]. To date, pattern recognition algorithms and deep learning models are employed as auxiliary aids to locate microcalcification lesions as well as support the detection of possible missing abnormalities, which generally improve the sensitivity of suspicious lesion detection.

Diagnosis is a systematic procedure comprising the evaluation of disease etiology, patient medical history, physical examination, diagnostic imaging, and laboratory results. With the integration of AI, computer-aided diagnosis (CADx) offers repeatable and reproducible outputs, such as staging (based on tumor, node, metastasis (TNM) classification [47]) and grading (based on Nottingham Histopathology Grading (NHG) system [48]) of breast cancer, via systematic processing of quantitative lesion information. CADx could also be extended to estimate prognostication in accordance with the detected ailment and predict the possible outcomes based on the given clinical treatments. Similar to radiomics, AI has a paramount role in interpreting imaging genomics which provides meaningful multivariate biological information (e.g., gene expression, chromosome copy number, somatic mutation, and relevant molecular significances). Correlating information from both of these emerging fields with the help of AI has great potential in complementing diagnosis output and synergistically leads to precision medicine.

Temporal monitoring of cancer has a paramount role in cancer management, especially in the assessment of treatment response and post-treatment follow-up. The present clinical routine in cancer monitoring using predefined metrics (e.g., tumor longest diameter) listed by the World Health Organization (WHO) and Response Evaluation Criteria in Solid Tumors (RECIST) provides limited information and is found insufficient to fully define the cancer status [49,50]. AI-based monitoring, however, can provide a better correlation of meaningful clinical data regardless of the complexity with improved capability in capturing subtle changes over time. Although a mature AI-based monitoring system is not yet available, the great utility and increasing roles of AI in cancer temporal monitoring are remarkable.

In every stage of cancer management, healthcare systems are generating multi-dimensional data creating and establishing themselves as big-data repositories. Big data are defined as a large amount of information that grows in three aspects: volume, velocity, and variety (known as 3 Vs), and that is unmanageable using conventional methods, such as software and/or internet-based platforms [51,52]. Big data are highly relevant in healthcare, especially in answering clinical questions about rarity and heterogeneity incidence, treatment patterns, and cancer prognostication. In recent years, the National Institutes of Health (NIH) has taken initiative toward establishing electronic health records by launching a program, namely “All of us” (https://allofus.nih.gov/) aiming to collect one million or more patient data over the next few years. Considering the roles of managing and analyzing big data retrospectively and predicting the potential outputs, AI is well suited to resolve these challenges and is invaluable with great utility in meeting the requirements.

## 2. Systematic Literature Search Methodology

### 2.1. Research Question

The primary research question of this umbrella review is: “What are the patterns, trends, quality, and types of reviews that documented AI applications in breast imaging over the years?” The research question is intentionally formulated in a broad manner to maximize the inclusion of review works describing AI applications in different breast imaging modalities.

### 2.2. Search Strategy

The umbrella review is intended to include review articles over the past decades. Thus, no date or year restriction was included in the search strategy. The systematic search was conducted in accordance with the Preferred Reporting Items for Systematic Reviews and Meta-Analyses (PRISMA) guidelines [53]. To identify the relevant review works, a structured search query was first formalized using the union of keywords in two primary facets: AI and breast imaging alongside a set of synonym terms (Table 1), aided with “AND” and “OR” operators. AI and breast imaging were used as the primary keywords to assure the inclusion of all umbrella terms corresponding to each keyword. For example, machine learning, deep learning, computer vision, data science, and automation are umbrella terms for AI. For example, all 20 breast imaging modalities are encompassed within the keyword: breast imaging. The comprehensive search was performed in databases, such as PubMed, Scopus, Web of Science, and Google Scholar. Once a relevant review work was identified, the references of the corresponding review work were screened to identify new relevant review works that had not been retrieved throughout the initial search. This process was iterated until no new relevant review works were found.

### 2.3. Study Selection

**Figure 4:**
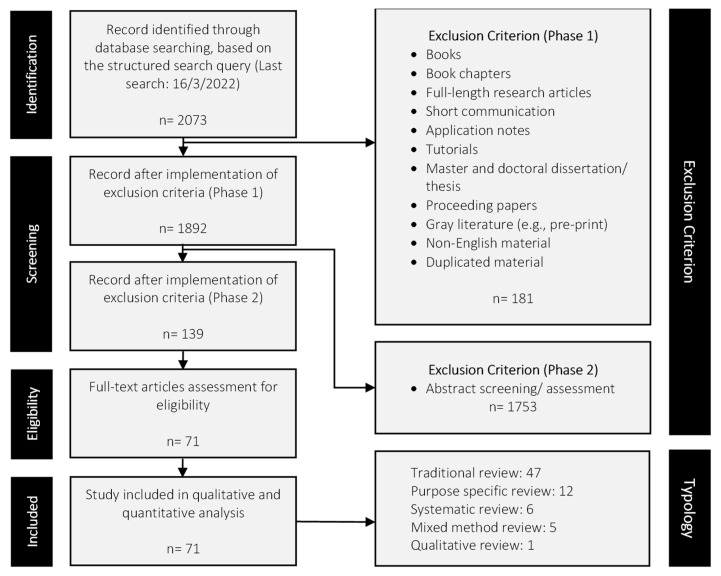
Study selection flowchart based on the PRISMA guidelines [53].

To assure the quality and acceptability of the included works, only peer-reviewed journal review articles were considered eligible. In Phase 1, books, book chapters, full-length research articles, short communication, application notes, tutorials, masters and doctoral theses, proceeding papers, gray literature (e.g., pre-print), non-English material, and duplicated material were excluded. The last search performed using the structured search query was on 16 March 2022. In Phase 2, abstract screening was first performed, and subsequently, the remaining review works were subjected to full-text screening. The primary inclusion criteria are: (1) the review works must describe the integration of AI and/or any subset of AI and (2) the review works must concentrate focus on any modalities (either one or more) within breast imaging. Review works that do not comply with the inclusion criteria were considered irrelevant and excluded. For example, review works reporting solely on applications of AI (or any subset of AI) in healthcare or documenting solely the application of imaging modalities within breast cancer is not the interest of this umbrella review. Figure 4 summarizes the PRISMA guideline alongside the exclusion criteria of this umbrella review. Overall, 2073 review works were retrieved from the initial search using the structured search query. In Phase 1, 181 review works were excluded, leaving 1892 articles. Abstract screening in Phase 2 eliminated 1753 review works, leaving 139 articles. After the full-text screening, 71 review works remained and these articles were included in the subsequent qualitative and quantitative synthesis activities.

### 2.4. Assessment of Reporting and Study Quality

Quality assessment in review works is crucial and reflects capability in preparing comprehensible and subjective reporting. Depending on the type of review, the quality of reporting in the included review works was assessed using appraisal tools, such as A Measurement Tool to Assess Systematic Reviews (AMSTAR) [54] and Scale for the Assessment of Narrative Review Articles (SANRA) [55]. These appraisal tools provide a checklist for the assessment and validation of reporting quality determining if the review works are comprehensive, equipped with added values to the reader, proper referencing, and assessing the likelihood of publication bias. AMSTAR is used to assess the methodological quality of systematic reviews, consisting of 11 components for content validity. Whereas, SANRA is used to assess the methodological quality of non-systematic reviews, which consists of six components. Depending on the type of review, each component within AMSTAR or SANRA was analyzed for each included work by two authors independently and answered with “Yes”, “No”, “Can’t answer”, and “Not applicable” (for AMSTAR) or given a score ranging from 0 (general low) to 2 (general high) (for SANRA). A third author is involved, if and only if discordances are raised in the assessment of reporting quality. As a result, the AMSTAR and SANRA results will be primarily used here to provide a summary of the reporting quality of the included works. For this umbrella review, similarly, the content validity, risk of bias, and applicability were assessed by two authors independently using the AMSTAR appraisal tool.

### 2.5. Data Extraction

To answer the research question, qualitative and quantitative data from the included review works were meticulously distilled in order to create a meaningful and functional summary endpoint. Qualitative data, such as the type of imaging modalities, AI techniques, and highlights of the included review work, were extracted, organized, compiled, and tabulated to offer a “one-stop center” synthesis. Quantitative data, such as bibliometric information and co-occurrence analysis, were collated and synthesized to offer a “bird’s eye” view of the topic of interest. The mineable data were systematized using graphics, charts, and tables. For each included review work, information such as author name and affiliation were manually disambiguated, e.g., “Lamb, Leslie R.”, “Lamb”, and “Leslie R. Lamb” refer to the same author; “Harvard Medical School” and “HMS” refer to the same affiliation. Still, subsequent quantitative synthesis activity using software such as VOSViewer will interpret them as independent authors or affiliations if a disambiguation step is not performed.

### 2.6. Data Reporting

The key findings of the umbrella review, including data analysis, qualitative, and quantitative synthesis were presented narratively using a descriptive approach. The analysis was supported with graphics, charts, and tables, aiming to highlight the key features and facilitate comprehension. Microsoft Excel 2019 was used for data compilation. VOSViewer 1.6.18 software for windows was used to perform bibliometric analysis [56]. The typology of the included review works was illustrated using a pie chart. The most contributing journals and publishers were illustrated using bar charts. Temporal scientometric data were illustrated using a line chart. Geographical scientometric data were illustrated using a world map chart. Subject area profiling was illustrated using a tree map chart. The quality assessment was illustrated using stacked bar charts, and co-occurrence analysis and keywords mapping were illustrated using bibliometric networks. A table was used to systematize the qualitatively mineable data (e.g., type of imaging modalities, AI techniques, and highlights of the included review work) from each included review work.

### 2.7. Threats to Study Validity

To minimize potential bias and assure study validity, in this review, a structured methodology was adopted: (1) Study selection bias: the search activity shall comply with the structured search query formalized in Section 2.2 and study selection criteria as in Section 2.3. (2) Data selection bias: two authors independently performed the search activity using the structured search query, followed by abstract and full-text screenings. Meetings and discussions were organized to resolve disparities raised at any phase. If necessary, a third author was engaged to draw the conclusion. (3) Functionality assessment: a self-assessment was conducted using the adapted appraisal tool, AMSTAR, to guide and assure the functionality of this umbrella review.

## 3. Results

To answer the primary research question of this umbrella review, qualitative and quantitative data are extracted from the 71 included review works and systematized into nine sub-sections, revealing the patterns (see Section 3.5, Section 3.6, Section 3.7 and Section 3.8), trends (see Section 3.2, Section 3.3 and Section 3.4), quality (see Section 3.9), and types of reviews (see Section 3.1) which documented AI applications in breast imaging.

### 3.1. Typology of the Included Review Works

**Figure 5:**
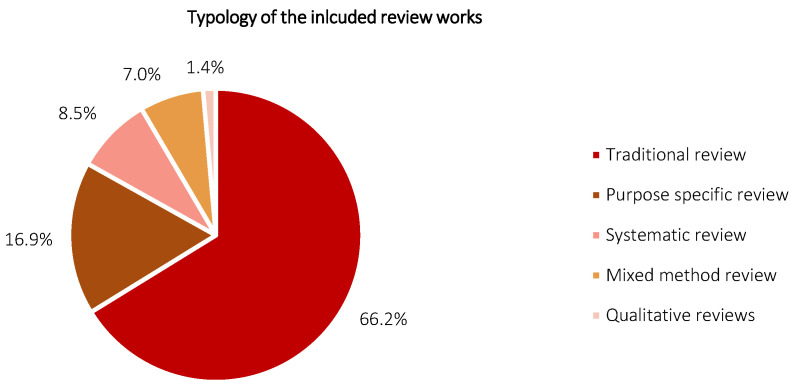
Typology of the included review works.

Figure 5 shows the typology of the included review works. The typology was assessed in accordance with the criteria stated in [26], and classified into the traditional review, purpose-specific review, systematic review, mixed-method review, and qualitative review. In brief, a traditional review aims to include extensive literature with efforts made to evaluate the quality of each included study, and to perform a high degree of analytic description alongside critical conceptual synthesis. A purpose-specific review focuses on identifying specific viewpoints, ideas, and/or concepts and targets to define attributes of the viewpoints. A systematic review employs a structured search strategy, encompassing a well-defined methodology or guideline and focusing in qualitative and quantitative synthesis activities. A mixed-method review offers a combination of various review methodologies but usually inclusive a systematic literature review (e.g., a combination of qualitative and quantitative review methodologies). A qualitative review, alternatively termed as experiential review, encompasses qualitative data synthesis popularized within a specific scope. According to Figure 5, the included review works constitute 66.2% (47 reviews), 16.9% (12 reviews), 8.5% (six reviews), 7.0% (five reviews), and 1.4% (one review) for traditional review, purpose-specific review, systematic review, mixed-method review, and qualitative review, respectively. The traditional review is found to be the most prevalent typology here, followed by purpose-specific review and systematic review. Mixed-method review and qualitative review are relatively scarce, corresponding to the body of the included works. The distribution of typology is speculated to be highly correlated to the popularity of review type amongst researchers in AI-dominant subject areas, such as computer science and engineering, where a remarkable rise in systematic reviews has been observed over the past five years (i.e., 2018: one review; 2021: five reviews). Traditional review and purpose-specific reviews both show a noticeable lack of well-defined methodology, which signals the need for structure guidelines as one of the research avenues. Notably, the qualitative data, e.g., imaging modalities, AI techniques, and highlights of each included review work, are systematized in Appendix A (Table A1) in accordance with the respective typology.

### 3.2. Distribution of the Most Contributing Journals

**Figure 6:**
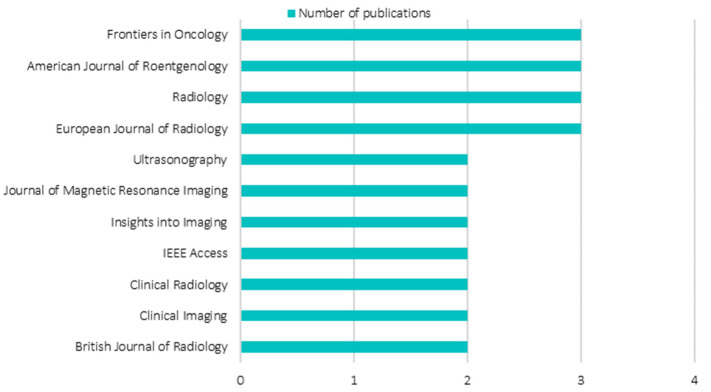
Analysis of most contributing journals.

Figure 6 shows the most contributing journals publishing review articles pertaining to AI in breast imaging. Considering the ubiquitous characteristic of the keyword: AI. In order to avoid ambiguity, only journals with at least two review article publications are included. According to Figure 6, journals with two review articles publication, each contributing 2.8% towards the whole, are Ultrasonography, Journal of Magnetic Resonance Imaging, Insights into Imaging, IEEE Access, Clinical Radiology, Clinical Imaging, and British Journal of Radiology. Whereas journals with three review article publications, each contributing 4.2% towards the whole, are Frontiers in Oncology, American Journal of Roentgenology, Radiology, and European Journal of Radiology. All the journals aforementioned, apart from IEEE Access which is a multi-disciplinary engineering journal, share highly similar aims and scope. These journals are dedicated to education and frontier strategies, typically in state-of-the-art reviews, opinions, and research articles in oncology, radiology, nuclear medicine, and imaging. Interestingly, over the years, amongst the most contributing journals, none were from AI-oriented journals, such as IEEE Transactions on Pattern Analysis and Machine Intelligence. It is reasonable to conclude that the review works in AI in breast imaging are heavily skewed towards the keyword: breast imaging, where AI technology is viewed as a rousing advancement that offers betterment in various aspects in the field of interest.

### 3.3. Distribution of the Most Contributing Publishers

**Figure 7:**
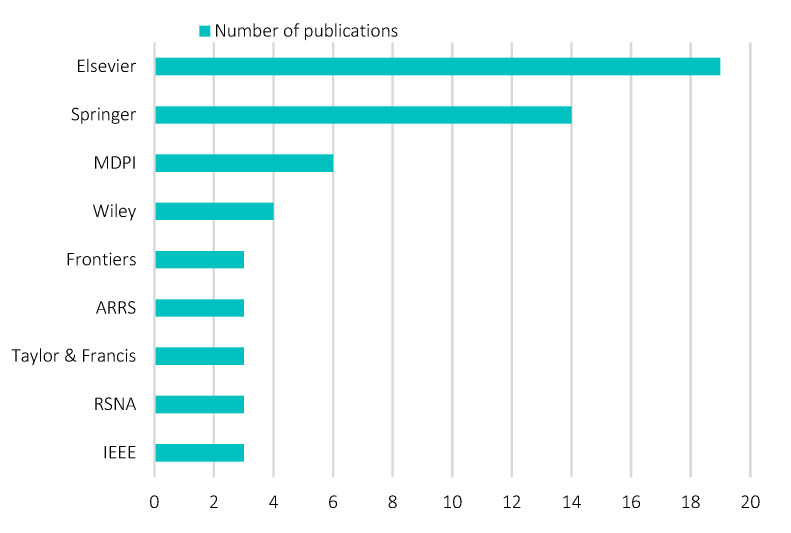
Analysis of most contributing publisher.

To assure an unbiased data selection, the structured search query was performed on general databases, such as PubMed, Scopus, Web of Science, and Google Scholar, in lieu of search engines provided by specific publishers, such as ScienceDirect, SpringerLink, and Multidisciplinary Digital Publishing Institute (MDPI). Figure 7 shows the most contributing (top five) publishers of review works pertaining to AI in breast imaging. Overall, 21 publishers were retrieved. Elsevier appeared to be the most contributing publisher on the topic of interest, contributing 26.8% (19 review articles), followed by Springer (19.7%, 14 review articles), MDPI (8.5%, six review articles), and Wiley (5.6%, four review articles). Frontiers, ARRS, Taylor & Francis, RSNA, and IEEE each contributed 4.2%, equivalent to three review articles. Overall, the publishers demonstrate an oligopoly relationship on the topic of interest, with predominance held by Elsevier and Springer. This finding is aligned with a previous study that focused on scientometric analysis publications sampled across multivariate topics from 1973 to 2013 [57]. The oligopoly relationship, typically between Elsevier and Springer in the global publishing industry, is speculated to remain similar across different topics. Evidence of this is found in a recent report analyzing bibliographical information of publishers [58] as well as in a recent systematic review in precision agriculture [59]. In recent years, pressure from various stakeholders has led to research transformation from closed access to open access. Open access journals are observed with an increase in normalized impact factor and average relative citations [60], which together are the elemental factors (statistically significant) influencing publication tendency amongst researchers [61]. Publishers, such as Elsevier, Springer, and MDPI, are making swift progress in transforming journals to open access. For example, in 2017, Elsevier indicated their intention to make the journal transition and work towards the principles behind the Sponsoring Consortium for Open Access Publishing in Particle Physics (SCOAP3) as well as to construct alternative access models tailored according to different geographical needs. For example, Springer is committed to accelerating the adoption of open access by complying with the institutional open access agreement. For example, MDPI was one of the pioneers in open-access scholarly publications as of 1996.

### 3.4. Temporal Scientometric Analysis

**Figure 8:**
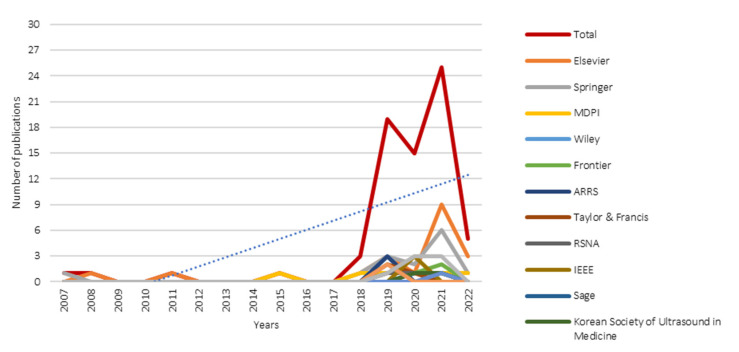
Temporal scientometric analysis of the included review works.

Figure 8 shows the temporal scientometric analysis of the included review works. As mentioned in Section 2.2, no date or year restriction was included in the structured search query. According to Figure 8, review articles complying with the structured search query could be traced back as early as 2007 (one review article), which was published in La Radiologia Medica, Springer. Despite significant progress in understanding the theoretical underpinnings of AI having begun in 1956, to incorporate in the medical domain, e.g., breast imaging, a number of validations and hurdles must be first overcome. Notable research interest in the publication of review articles is observed only after a decade (i.e., 2018) from the first review article in 2007. This observation could be attributed to the increased reliability and utility of AI across multiple non-medical spectrums, indirectly gaining trust and level of acceptance of AI reliability amongst medical experts. In the ever-increasing demand for quality healthcare, the “one-size-fits-all” approach is no longer valid. Progress in medical domains, such as breast imaging, is moving towards precision medicine. In recent years, researchers have actively sought rigorous clinical proof of AI utility in breast imaging and progressively made concerted attempts to push AI technology from pilot tests to validation in clinical trials, aiming to apply it as a fundamental segment in the standard clinical protocol. The blue dotted line in Figure 8 shows the overall publication trendline. Overall, a linear increment is observed across 15 years (i.e., 2007 to 2022).

### 3.5. Geographical Scientometric Analysis

**Figure 9:**
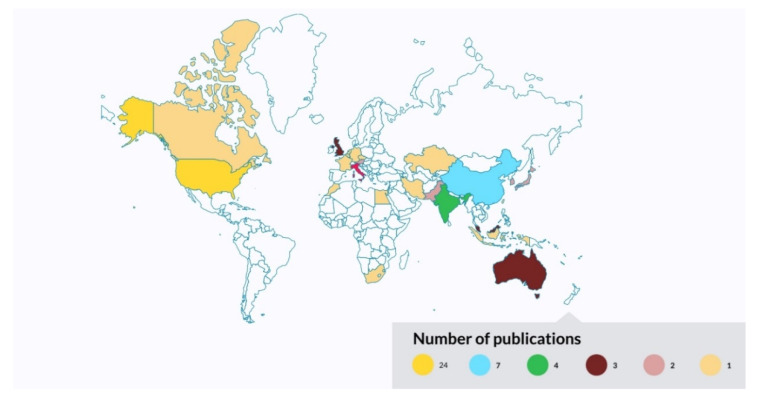
Geographical scientometric analysis of the included review works.

Figure 9 shows the geographical scientometric analysis of the included review works. For each review article, only the geographical information of the corresponding author is retrieved. For articles with more than one corresponding author, each geographical information is allowed to contribute once to the scientometric analysis. According to Figure 9, the United States appeared to be the most contributing country summarizing research works pertaining to AI in breast imaging as of 2014, corresponding to 33.8% (24 review articles) of the entire body of literature. This is followed by China (contributing 9.9%, seven review articles); India, Italy, and the United Kingdom (each contributing 5.6%, four review articles); Australia (contributing 4.2%, three review articles); and Austria, Japan, Korea, Malaysia, and Pakistan (each contributing 2.8%, two review articles). 

**Figure 10:**
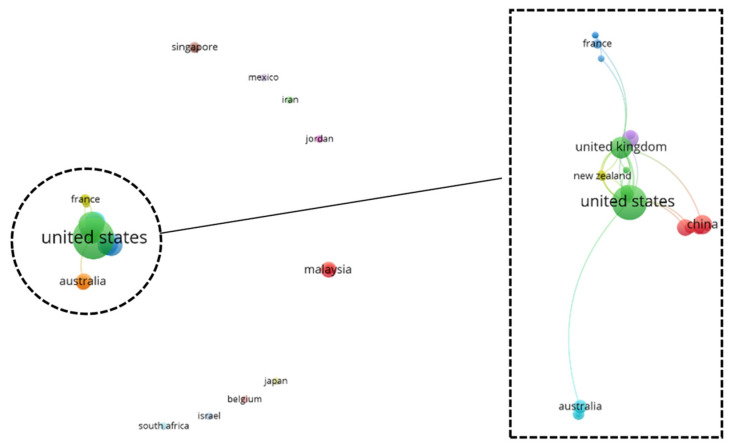
Co-authorship bibliographic network analysis: country.

**Figure 11:**
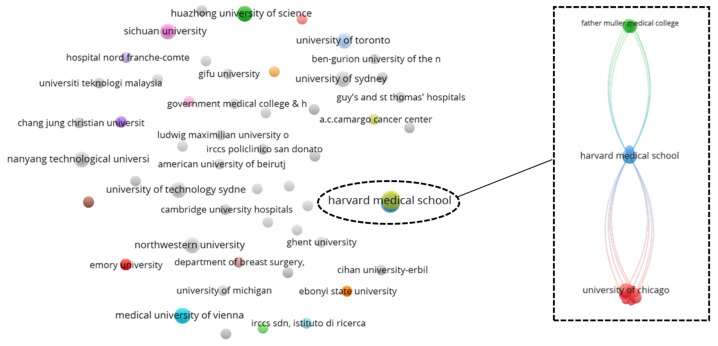
Co-authorship bibliographic network analysis: affiliation.

**Figure 12:**
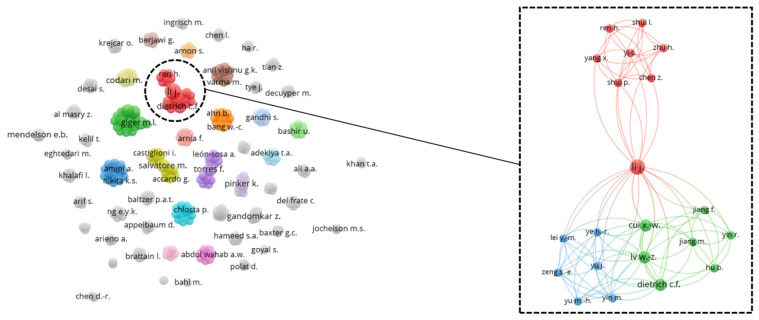
Co-authorship bibliographic network analysis: author.

To gain a holistic overview of the captured geographical information, a top-down co-authorship bibliographic analysis is performed, analyzing three aspects on a cascading level: country (Figure 10), affiliation (Figure 11), and author (Figure 12). Co-authorship is a commonly used operational proxy in studying research collaboration (researcher oriented) as of 1980 [62]. Briefly, in Figure 10, Figure 11 and Figure 12, each item is represented using a label and a node. The size of the label and node reflects the weight of the specific item, such that the higher the weightage, the larger the label and node. The color of the node reflects a local cluster, connected using lines that are termed links. The shorter the links, the stronger the relatedness of both items. 

According to Figure 10, the United States has the strongest link strength across 42 countries, attaining a value of 21, followed by the United Kingdom (total link strength of 17), Malaysia, Netherlands, and New Zealand (each with a total link strength of six). There are 15 local clusters found in the country’s co-authorship network. Interestingly, nine small local clusters were found isolated from one another, forming a local network with fewer than two countries. The six connected clusters are highlighted in Figure 10. The United States has a strong relationship with countries, such as the Netherlands, Canada, and the United Kingdom. This could be attributed to efforts by government agencies, such as the National Institute of Health (NIH), in promoting medical research and supporting scientific studies in the medical division. 

According to Figure 11, the Harvard Medical School appeared as the affiliation with the strongest total link strength of 15, a predominance across 162 affiliations. A total of 52 local clusters are found in the affiliation co-authorship network, such that 49 local clusters are isolated from one another. The remaining three connected clusters are highlighted in Figure 11. Amongst the connected clusters, Harvard Medical School has the highest connectivity weightage with a close relatedness to MIT Lincoln Laboratory, forming a small local cluster with only two affiliations. 

According to Figure 12, the author, namely Li J. has the strongest total link strength with a value of 23, followed by Giger M.L., Gillies R.J., and Schabath M.B., each with a total link strength of 19. A total of 327 authors are found in the author co-authorship network, forming 54 clusters. Amongst the clusters, only three clusters are inter-connected and are highlighted in Figure 12. 

It is undeniable that research collaboration greatly improves scientific findings and jointly concerted efforts in resolving complex engineering problems. International collaboration for AI integration in breast imaging is below expectations, however. Based on the network analysis, in a broad view, a high number of isolated local clusters reflects a low rate of international collaboration on the three cascaded levels, which indicates the need for efforts in nurturing multinational collaboration, establishing global resources, as well as knowledge sharing for the mutual good in resolving world health issues.

### 3.6. Bibliographic Coupling Network Analysis: Country

**Figure 13:**
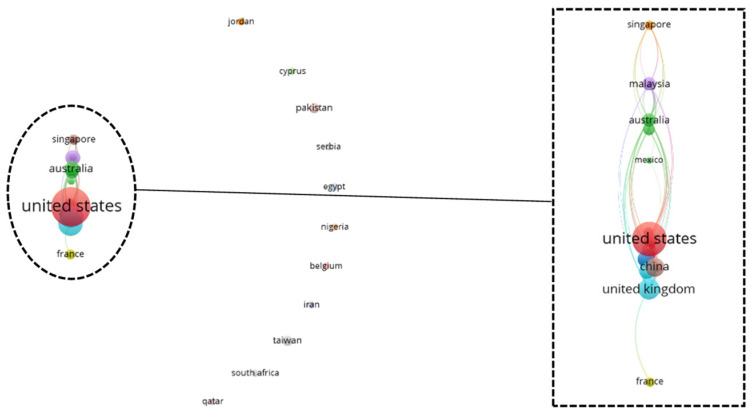
Bibliographic coupling network analysis: country.

Bibliographic coupling, like co-authorship, however publication oriented, inherits the benefits of recommending relevant works. When a common work is cited as a reference in two documents, they are thus bibliographically coupled [63]. Here, a strong total link strength reflects a high number of common works cited as references in the respective review article. Figure 13 shows the bibliographic coupling network, analyzed at the country level. According to Figure 13, the United States has the strongest total link strength, with a value of 646, followed by the United Kingdom (total link strength: 630), China (total link strength: 357), Australia (total link strength: 329), and South Korea (total link strength: 328). A total of 21 clusters are found across 42 items, such that 13 local clusters are isolated from one another. The eight connected clusters are highlighted in Figure 13. Notably, the top five countries with the strongest total link strength are within the eight connected clusters, alongside other remarkable items (e.g., Singapore, Malaysia, Italy, and France), creating local networks and subsequently local clusters.

### 3.7. Subject Area Profiling

**Figure 14:**
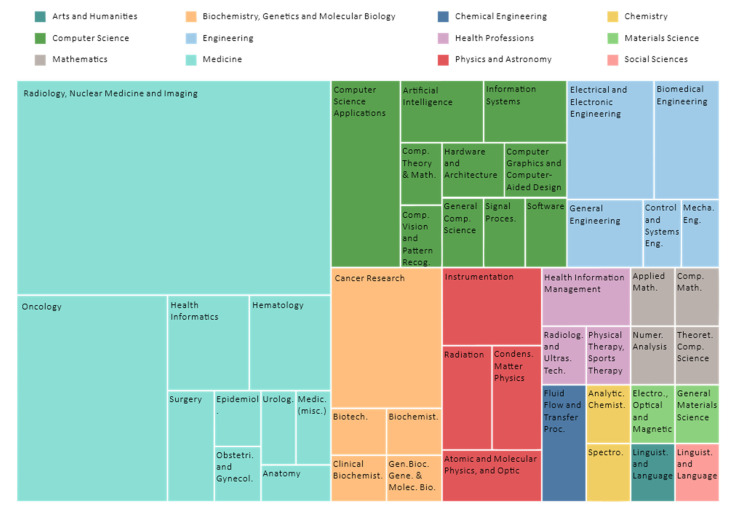
Subject area profiling.

Figure 14 shows a tree map profiling the subject areas of the included review works, distilled across 56 journals in accordance with the Scopus database. Importantly, journals can be indexed in one or more subject areas, positioned in accordance with the aims and scope of the journal. For example, CA: A Cancer Journal for Clinicians is a single subject area, and a medical research-oriented journal focuses only on medicine. For example, Sensors is a multi-disciplinary journal indexed across five distinct subject areas: Physics and Astronomy, Engineering, Computer Science, Chemistry, as well as Biochemistry, Genetics, and Molecular Biology. According to Figure 14, most of the journals are within the subject areas of Medicine (76.8%, 43 journals), followed by Computer Science (21.4%, 12 journals), Engineering (17.9%, 10 journals), Biochemistry, Genetics, and Molecular Biology (17.9%, 10 journals), and Physics and Astronomy (10.7%, six journals). The subject areas collated are plausible and aligned with the finding in Figure 6, such that the most contributing journals pertaining to AI in breast imaging are within the medical division and are biased towards the keyword: breast imaging.

### 3.8. Keywords Co-Occurrence Network

**Figure 15:**
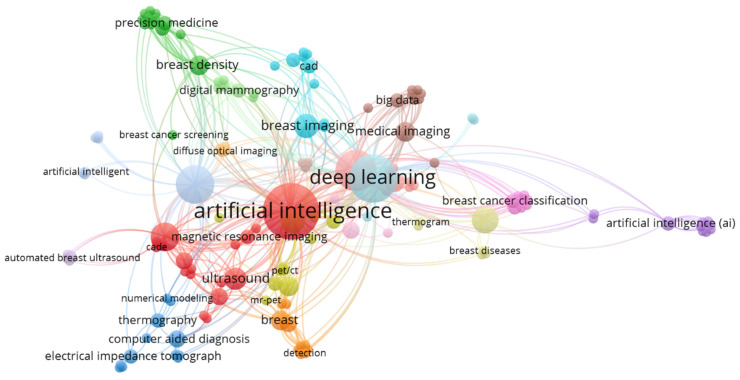
Indexed keywords co-occurrence mapping.

Figure 15 shows the keywords co-occurrence network distilled from the included review articles, which impart the trend and focus of the scientific interest within AI in breast imaging over the past decades. The network constitutes 150 items, creating 18 local clusters, and is dominated by AI and its umbrella terms. AI, deep learning, and machine learning exhibit the strongest total link strength with values of 143, 112, and 91, respectively, followed by breast cancer (total link strength: 76) and mammogram (total link strength: 38). According to Figure 15, although various adjunctive breast imaging modalities (e.g., ultrasound, MRI, thermography, PET, and CT) have been studied every so often, mammogram, however, remains the focal point for AI integration. From a broader perspective, Figure 6 and Figure 15 together reveal the researchers’ proclivity towards the publication of their review works, such that the options of journals and keywords are respectively breast imaging and AI-oriented.

### 3.9. Quality Assessment of the Included Review Works

**Figure 16:**
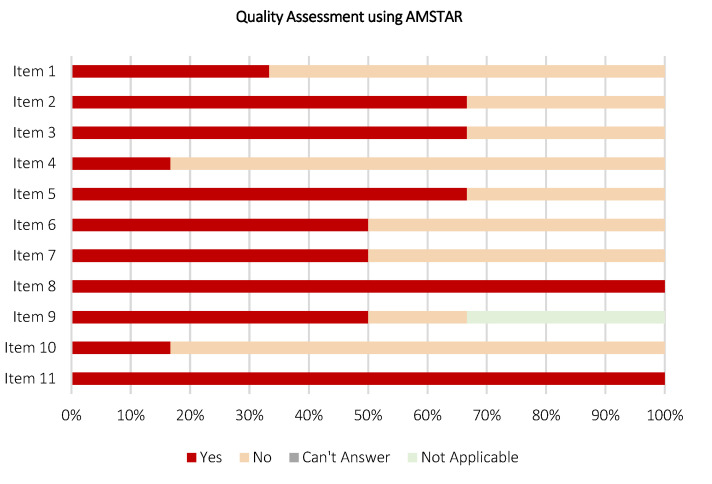
Quality assessment on six included review works (systematic review) using AMSTAR appraisal tool.

**Figure 17:**
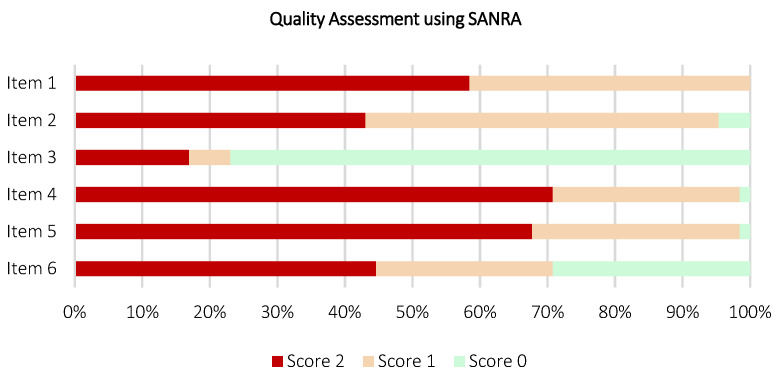
Quality assessment on 65 included review works (non-systematic review) using SANRA appraisal tool.

Figure 16 and Figure 17 depict the stacked bar charts for quality assessment of the included review works, constituting six systematic reviews and 65 non-systematic reviews using AMSTAR and SANRA appraisal tools, respectively. According to Figure 16, on AMSTAR adherence, a majority of the included systematic reviews scored “yes” ratings on eight out of 11 AMSTAR components. Only two included systematic reviews were rated “yes” for Item 1 [64,65] and one systematic review [64] was rated “yes” for both Items 4 and 10. Overall, the included systematic review works demonstrate promising relevance, capable to extract and synthesize data from the available evidence, with a guided methodological reporting procedure to formalize findings. Nonetheless, a low number of reviews found reporting data in Items 1 (“priori” design assessment), 4 (inclusion criteria assessment), and 10 (publication bias assessment) flagged the need for extra priority in reporting these items as part of the future research avenue. According to Figure 17, on SANRA adherence, most of the included non-systematic reviews attained “score 2” ratings on four out of six SANRA components. For Item 2 (aims and formulation of questions assessment), 28 reviews were rated as “score 2”, alongside 34 and three reviews were rated as “score 1” and “score 0”, respectively. For Item 3 (literature search assessment), only 11 reviews were rated “score 2”, followed by four, and 50 reviews were rated as “score 1” and “score 0”, respectively. As aforementioned, the non-systematic reviews have a noticeable lack of well-defined methodology. Here, the quality assessment outcomes further support this statement, reflected by a low number of data reports in Items 2 and 3, which specifically assess the structure of review methodology, from the research question formalizing and data sourcing perspectives. The full quality assessment scoring tables for each included review work using AMSTAR and SANRA are available in Appendix A (Table A2 and Table A3, respectively).

## 4. Self-Assessment, Limitations, Challenges, and Future Direction

### 4.1. Self-Assessment

The present study focuses on the scientometric analysis of an umbrella review on the topic pertaining to AI in breast imaging over the past decades. Structured review methodological procedures were adopted to collate and formulate findings revealing patterns, trends, quality, and types of the included review works in the topic of interest. The search strategy, publication threat, and methodologies, such as PRISMA guidelines, were adopted and detailed in Section 2. AMSTAR appraisal tool was used to study self-assessment in view of the face and content validity (Table 2). According to Table 2, the present study scored “yes” for all 11 AMSTAR components. To support the self-assessed AMSTAR rating, justifications were given for each AMSTAR component, as recommended by [66]. Here, within review works, the authors advocate the inclusion of study self-assessment as a fragment of the structured review methodological procedures. Quality assessment is a crucial instrument to assure the relevancy of the review work toward the topic of interest while avoiding possible bias and examining the face and content validity. 

### 4.2. Limitation

The findings of this umbrella review are subjected to several limitations. First, only review works made available in full text using the English language were included for the subsequent synthesis activities. This could possibly introduce bias to the analysis outputs, describing the patterns, trends, quality, and types of review work within the dedicated scope. Second, as detailed in Section 2.3, review articles solely describing AI application (or any subset of AI) in healthcare or solely reporting the application of various breast imaging modalities were not within the interest of this umbrella review. As a result, review articles that offer emerging AI with potentially useful insights and utility in breast imaging may be excluded. Third, review works that were not populated by databases such as PubMed, Scopus, Web of Science, and Google Scholar were not included in the synthesis activities. 

Moving toward precision medicine, AI finds great utility in assisting medical experts in every stage involving clinical decisions. As technology continues to develop in concert with advances in engineering, mathematics, and computer science, AI has emerged as one of the primary research areas at rapid rates. Although patterns, trends, and progress pertaining to AI in breast imaging are expected to evolve in the near future, the scientometric findings of this umbrella review continue to be viable and reflect the current state of research globally over the past decades. The umbrella review complies with a comprehensive search strategy using structured methodology such as PRISMA (Section 2.2), delineates study inclusion criteria (Section 2.3), and scrutinizes the threats to study validity (Section 2.7), alongside a self-assessment using AMSTAR.

### 4.3. Challenges and Future Direction

Over the years, AI has been advocated in breast imaging as a potential game-changer to meet the long-standing, constantly rising demand for high-quality healthcare, typically in precision medicine. Despite all of the reported successes, a number of obstacles and bottlenecks must be surmounted prior to the widespread clinical adoption. Breast imaging procedure generates medical images for diagnosis purposes and is rarely curated with labels, demarcation, and delineation of cancerous, non-cancerous, and microcalcification regions. Data curation is exorbitant in terms of both time and cost, requiring meticulous effort from experienced medical experts. This remains the core challenge in automating standard clinical procedures, exacerbating the adoption of data-hungry AI in cross-case referencing. Although methods, such as semi-supervised [67] and unsupervised [68] algorithms, could potentially alleviate this challenge, other barrages, such as data imbalance, are inevitable. Considering the plethora of breast imaging modalities, standardization in the ground truth and benchmarking is crucial for generalizability purposes in AI, typically across different ground truth providers. To date, within breast imaging, the volume of scientific-rich resources made available for training purposes is encouraging [69]. However, the accessibility and amount of curated data are in a stagnant paradigm [70]. Concerted efforts from various parties, including government, clinical institutions, and universities, should be encouraged to foster the integration of AI in breast imaging. 

From a regulatory standpoint, the FDA has regulated AI-assisted automated systems since the 1990s [71]. The accuracy, robustness, and generalizability of AI systems are enduring concerns. The AI systems are expected to cogently demonstrate consistent and persistent applicability before obtaining clearance from the respective regulatory bodies. As aforementioned, healthcare systems are now establishing themselves as big-data repositories. Running AI architectures, such as neural networks, across big data would profoundly offer comprehensive cross-data/case referencing. This, however, flagged data privacy and security concerns. Compliance with the data privacy and security acts necessitates solutions, such as cryptonets [72], where homophobic encryption allows the neural networks to perform data training over encrypted data. AI has challenged the patent system in light of patent law, which presumes that inventors are human [73]. If the intellectual property (IP) resultant from AI, which could be life-saving and beneficial within the medical imaging context, is not patentable, investors may lose interest or be less incentivized to fund projects with AI-oriented inventors leading to nationwide depletion of AI research.

Miseducated AI algorithms could violate ethical standards, corrupt morality, signal ethical dangers, and cause severe harm, in addition to aggravating societal medical pressure [74,75]. For example, when using cost as a proxy for healthcare requirements, AI falsely recognized that black patients were healthier than white patients who equally suffered from the same sickness, as fewer medical costs were spent on black patients [76]. For example, white patients were measurably given better care than black patients who were in comparable situations [77]. The AI algorithm could be unethical by design, mirrors unconscious racism, thoughts, and biases within the developers, and is further complicated and exacerbated by the tension between opting for optimal healthcare or medical profit [77,78]. Moreover, the AI algorithm is inherently objective in nature, with characteristics acquired from historic training data representing specific patient cohorts. Thus, under-represented cohorts may be afflicted by inaccurate diagnoses. The fairness of AI must be given priority. Adding more data and careful parameter fine-tuning may not be plausible as vulnerable groups, especially the under-resourced cohorts would be unrepresented. 

To date, AI systems are likely to exhibit a “black box” characteristic, which lacks interpretability, explainability, and transparency, which, in totality, hinders trust in AI systems [9,79]. Trust in AI systems is crucial, reflecting the user’s level of acceptance of the clinical outcome provided by the AI systems [80]. The AI systems in breast imaging should be equipped with scientific reasons behind a clinical outcome and help the user decipher the underpinning factors. To keep healthcare affordable, AI should not be meant to prevent medical experts from making occasional mistakes, but to fully automate certain procedures that are currently performed by medical experts. Ultimately, AI in breast imaging should increase clinical productivity while avoiding unnecessary increases in healthcare costs.

Although AI can detect ailments and auxiliary findings that may be clinically beneficial, these findings may also be clinically irrelevant. During the clinical transition stage, the findings from AI shall be carefully distilled and interrogated by medical experts, evaluating relevance to avoid unnecessary increases in patients’ tension, healthcare costs, and unwanted side effects while improving AI robustness and applicability. Over time, as AI systems attain maturity, the findings from AI could be incorporated as part of the standard reporting procedure for diagnostic and prognostic purposes. Medical experts can now perform continuous temporal assessment and quality assurance on the AI systems, such as AI quality improvement (AI-QI), as suggested in [81].

AI in breast imaging is a complex problem, complicated and embodies cross-disciplinary collaboration and cross-topic knowledge. To ensure the long-term success of AI integration within breast imaging, it is imperative that experts from diverse scientific backgrounds work together. Recently, convergence science has emerged as one of the research models for tackling such complex challenges [82,83]. Convergence science is an active area, and its definition is evolving over time. Nonetheless, a general consensus has been reached such that convergence science is not simply a set of experts meeting one another, but over time, the experts absorb knowledge from one another, breakthrough the respective silos of knowledge, reshape research mindsets, complement respective research experiences, and ultimately foster the development of new solutions [82].

From a broader perspective, breast imaging should not be an isolated measure of cancer. Core and incidental reporting on measures, such as molecular signatures, biomarkers, the nature of cancers, environmental factors, socioeconomic status, and social network settings, shall be considered. AI in breast imaging should be seen as one of the fundamental measures, alongside other computer-aided systems (e.g., wearable sensors and medical expert systems), to be integrated for the improvement of outcome predictions in both breast cancer diagnostic and prognostic stages.

Widespread AI adoption in breast imaging is complicated by multiple factors, and this issue contemplates the acquisition of funding, leadership, sustainable mechanisms, and awareness among medical practitioners. Adequate funding is not always available; and even if such funding exists, the funding utilization is likely suboptimal with ineffective practices across multiple sectors. The right leadership is required to advocate for, manage, and oversee the implementation of AI in breast imaging. To promote the widespread adoption of AI in breast imaging regardless of geography or socioeconomic status, policies, procedures, and guidelines must be developed and nationwide infrastructure (e.g., computer systems and internet access) must be evaluated.

## 5. Summary

While algorithms across a broad spectrum of breast imaging applications have demonstrated potential, AI has emerged as the dominant research paradigm, with compelling utility converging towards precision medicine and automatic triage. Integration of AI in breast imaging is flourishing and is anticipated to continue. In this formal scientometric umbrella review, the authors examined the patterns, trends, quality, and types of the included review work from the past decades in an effort to provide a concise but comprehensive overview of the topic of interest. This scientometric umbrella review functions as a scientific, scholarly communication, evaluates the level of consensus, identifies research gaps, highlights challenges, and provides commentary on the future direction of the field. For newcomers to the field, the scientometric umbrella review provides a holistic and timely overview, offers valuable insight into the intellectual landscape, understanding the development of literature, and highlights the road ahead. For existing experienced researchers, the scientometric umbrella review serves as an instrument for keeping their knowledge updated, especially in identifying research areas that are potentially relevant but beyond their immediate research topic. For relevant stakeholders, the scientometric umbrella review can be used to prioritize research and project funding to benefit areas that require impactful and immediate solutions through AI integration within breast imaging.

## Figures and Tables

**Table 1:** Search string.

Operator	Dimensions	Keywords, Synonyms, and Alternative Terms
AND	AI	artificial intelligence OR AI OR machine learning OR deep learning OR expert system
	Breast imaging	breast imaging OR breast imaging modality

**Table 2:** Study assessment on this umbrella review using AMSTAR appraisal tool.

Item	Description	Rating	Justification
1	Was an ‘a priori’ design provided?	+	Yes, the research questions and inclusion criteria are provided in Section 2.1 and Section 2.3, respectively
2	Was there duplicate study selection and data extraction?	+	Yes, excluded as detailed in Figure 4
3	Was a comprehensive literature search performed?	+	Yes, as detailed in Section 2.2
4	Was the status of publication (i.e., grey literature) used as an inclusion criterion?	−	No, inclusion criterion is provided in Section 2.3
5	Was a list of studies (included and excluded) provided?	+	Yes, provided in Appendix A
6	Were the characteristics of the included studies provided?	+	Yes, provided in Appendix A
7	Was the scientific quality of the included studies assessed and documented?	+	Yes, as detailed in Section 3.9
8	Was the scientific quality of the included studies used appropriately in formulating conclusions?	+	Yes, the scientific quality of the included review works from different perspectives were considered (Section 3). Recommendations and future direction are provided in Section 4.3
9	Were the methods used to combine the findings of studies appropriate?	+	Yes, as detailed in Section 2.6
10	Was the likelihood of publication bias assessed?	+	Yes, as detailed in Section 2.7 and Section 4.2
11	Was the conflict of interest stated?	+	Yes, as detailed in the Conflicts of Interest Section

+: Yes; −: No.

## Appendix A

Table A1 shows the qualitative data distilled from each included review work. Table A2 and Table A3 show the AMSTAR and SANRA rating for each included review work, respectively.

**Table A1:** Qualitative data distilled from each included review on imaging modalities, AI techniques, and the corresponding review highlights.

No.	References	Imaging Modalities	AI Techniques	Cited By *	Highlights
Traditional Review					
1	[84]	Thermography	CNN	21	Summarize the recent progress in breast cancer detection based on optical imaging (i.e., thermography) CNNs Application of deep neural networks for breast thermogram classification Future works in development of datasets, feeding the segmented image, assigning good kernel, and construction of a lightweight CNN model to enhance the CNN performance
2	[85]	Thermography	ANN: RBFN, KNN, PNN, SVM, ResNet50, SeResNet50, V Net, Bayes Net, CNN, C-DCNN, VGG-16, Hybrid (ResNet-50 and V-Net), ResNet101, DenseNet, and InceptionV3	11	Comprehensive review on related progress with AI in optical imaging (i.e., thermography) Highlight the contributions and limitations of each study Identify research gap Identify bottlenecks of ANN in breast thermography
3	[86]	X-ray, CT, ultrasound, MRI, nuclear, and microscopy	Deep learning	38	Summarize different medical imaging technologies Highlight the need of efficient medical data management techniques Summarize the advent algorithms for disease classification and organ/tissue segmentation using AI methods
4	[87]	Mammogram, ultrasound, and MRI	Machine learning and deep learning	1	Summarize the AI application in mammogram, ultrasound, and MRI Discuss the challenges and road ahead of AI in medical imaging
5	[88]	Mammogram, ultrasound, MRI, FDG PET/CT	Augmented intelligence and machine learning	16	Comprehensive overview on augmented intelligence in different breast imaging modalities
6	[89]	Mammogram, ultrasound, MRI,	Machine learning, CADe, ANN, CNN, and NLP	36	Discuss the potential of AI in breast imaging Identify the limitations and bottlenecks of AI application
7	[90]	Mammogram and ultrasound	Machine learning	1	Summarize the essential elements in data engineering, typically preparation of data for machine learning application Comprehensive review on databases, perspective, data integrity, and characteristic of a good data in the context of machine learning application
8	[91]	Thermography and electrical impedance tomography	Machine learning and CADe	15	Summarize the background, technical characteristics of machine learning techniques and CADe in thermography and electrical impedance tomography Identify the methods to enhance global performance Highlight recommendations for 3D breast simulation, pre-processing techniques, as well as relevant biomedical modality that has the potential in tumor localization and tumor size prediction
9	[92]	Transillumination imaging, diffuse optical imaging, and near-infrared spectroscopy	Machine learning	13	Comprehensive review on applicability (i.e., diagnostic ability and practical deployment) of the new spectroscopy techniques in discrimination of normal and cancerous tissue
10	[93]	Mammogram, Tomosynthesis, ultrasound, DBCT, MRI, DWI, CT, NIR fluorescence, and SPECT	SVM, ANN, and robotics	6	Overview on past and present AI methods in different breast cancer diagnostic imaging modalities
11	[94]	PET and MRI	Fuzzy logic and neural network	26	Summarize recent progress, key strengths, and weakness of PET/MRI in cancer imaging
12	[9]	Mammogram, tomosynthesis, DCE-MRI, and ultrasound	CADe and CADx systems	449	Comprehensive state-of-the-art review on AI approaches in medical imaging for four tumor types: lung, brain, breast, and prostate Identify the common clinical problem in medical imaging
13	[95]	MRI	Analytical radiomic-based (human-engineered) and deep learning-based CADe	44	Discuss the goal of AI in the context of breast cancer imaging, typically in MRI Review on current progress, potential application, and limitations of AI in breast MRI
14	[96]	MRI	Machine learning and deep learning	4	Discuss the current progress and future perspective of AI in breast MRI Highlight the importance of developing quantitative imaging biomarkers for precision medicine Discuss potential of machine learning and deep learning in breast MRI Discuss the future challenge of deep learning in the context of breast MRI and recommend AI-augmented clinical decision techniques
15	[97]	MRI	3D printing, augmented reality. Radiomics, and machine learning	12	Discuss the role of breast MRI in detection of DCIS with the aid of AI approaches
16	[98]	Mammogram, ultrasound, and MRI	ANN, CNN, CADe, and GANs	0	Comprehensive review on insight, impact, and role of AI in breast imaging
17	[99]	Mammogram	Machine learning (supervised, unsupervised, reinforcement, and deep learning)	6	Highlight and discuss key terminology, concepts, and common AI models in breast imaging
18	[100]	Mammogram and DBT	Deep learning	89	Comprehensive review on current progress and head ahead of the AI-based clinical application in mammogram, DBT, and radiomics
19	[101]	Ultrasonography	CNN	9	Current progress and potential application of AI models in breast ultrasonography
20	[102]	Mammogram, ultrasound, and MRI	Deep learning and CADe	32	Comprehensive review on state-of-the-art deep learning methods in mammogram, ultrasound, and MRI Discuss the challenges and bottlenecks which may impinge the advancement and integration of AI in clinical imaging workflow
21	[103]	Mammogram	Machine learning and radiomics	19	Discuss the CEM technique, screening, and diagnostic uses, and future applications with AI and radiomics
22	[104]	Mammogram, sonography, MRI, and image-guided biopsy	Deep learning and radiomics	2	Recent progress and potential value of digital analysis in breast imaging
23	[105]	Mammogram, ultrasound, PET, and MRI	ANN, SVM, and radiomics	1	Discuss key concept, state-of-the-art studies, and potential clinical use cases in breast imaging
24	[106]	Ultrasound	Deep learning	5	Discuss recent progress of deep learning in ultrasound imaging Discuss the workflow enhancement, typically on view recognition, image quality assessment, scanning guide, and quantification Recommend future perspective of deep learning in imaging workflow
25	[107]	Mammogram and ultrasound	Eye tracking tool and CADe	10	Highlight the importance of visual search in breast imaging Identify the underlying reasons for diagnostic error in breast imaging
26	[108]	Mammogram	CADe, CADx, machine learning, deep learning, and CNN	48	Review of recent progress of AI approaches in breast imaging Commentary on AI in medical diagnostic imaging
27	[109]	Mammogram and tomosynthesis	Deep learning	0	Synthesis recent progress of AI in mammography analyzing the risk in breast cancer Highlight the key priority, challenges, and future prospective of AI application in clinical workflow
28	[110]	Nuclear medicine	Deep learning and radiomics	1	Introduce the basic design of neural network Review of influential works, strengths, and limitations of deep learning approaches as compared to the conventional approaches
29	[111]	MRI, CT, PET, SPECT, ultrasound, tomosynthesis, and radiology	Neural network, deep learning, and machine learning	227	Discuss the basic terms of AI (e.g., machine and deep learning) and analyze the integration of AI in radiology
30	[112]	Mammogram, ultrasound, MRI, and tomosynthesis	ANN, CADe, CADx, CNN, deep learning, and machine learning	8	Overview on current application of deep learning approaches in breast radiology Identify and highlight the challenges and research gap of AI application in breast radiology
31	[113]	Mammogram, ultrasound, and MRI	Deep learning	5	Review of current knowledge and future prospective of AI-enhanced breast imaging modalities in clinical workflow
32	[114]	Mammogram, ultrasound, MRI	SNN, SDAE, DBN, and CNN	2	Review on state-of-the-art AI approaches in the last decade to detect breast cancer using different breast imaging modalities
33	[115]	Mammogram, ultrasound, MRI, and tomosynthesis	Deep learning and AI-CADe	79	Review the current limitations and future prospective of AI-CADe in breast imaging
34	[116]	Tomosynthesis, CT, and FDG PET/CT	ANN, DNN, SVM	84	Review the recent progress and strength of AI applications in cancer diagnostic and prognostic Identify how AI technology can improve cancer diagnostic and prognostic from the clinical perspective
35	[117]	Ultrasound	Machine learning and deep learning	8	General overview on the context of AI, machine learning, and deep learning technologies Review of current progress in deep learning technologies and highlight future perspective, challenges, and research gap of biomedical AI systems in ultrasound
36	[118]	Mammogram, tomosynthesis, ultrasonography, and MRI	CADe, radiomics, IoT, and machine learning tools	17	Critical review on advent methods for screening, diagnostic, staging, grading, molecular, and genetic biomarkers Highlight the interdisciplinary key terms of such methods for scientists and physicians
37	[119]	Mammogram and CT	Deep learning, CADe	6	Highlight major areas in oncology imaging with great potential of AI application Identify potential bottlenecks of AI application in oncology imaging
38	[120]	Mammogram, ultrasound, PET, CT, and MRI	CADe, ANN	127	Outline the progress of AI and CADe in general clinical imaging
39	[121]	Ultrasound	CADx	20	Highlight recent progress in CADx in breast ultrasound Recommend future direction of CADx in breast ultrasound
40	[122]	Mammogram, ultrasound, MRI, and thermography	Machine learning, deep learning, and CADx	42	Highlight new application of deep learning and machine learning approaches in detection and classification of breast cancer and outline the overview of such progress Review different breast imaging modalities and correlate these modalities with AI
41	[123]	Mammogram	Radiomics	0	Highlight the potential role of radiomics in mammography Identify the bottlenecks and research gap of such application
42	[124]	MRI and DCE-MRI	Radiogenomics	0	Review multidimensional mining algorithms in the context of MRI radiogenomics for CADe and CADx of breast tumors
43	[125]	Tomosynthesis, MRI, ultrasound, MBI	Machine learning and CADx	3	Review the current progress in imaging of high-density breast for diagnostic of breast lesions
44	[126]	Thermography	SVM, ANN, and CADx	42	Review of recent advances, recommendation, and road ahead for detection of breast cancer using infrared thermography
45	[127]	Mammogram	AI-CADe	62	Review of past, present, and future role of AI in medical imaging Highlight the challenges of AI application in imaging Highlight the role of AI-CADe with PACS
46	[128]	Mammogram and MRI	CNN	1	Highlight the fundamental in AI and its application in medical imaging analysis
47	[70]	General breast imaging	Machine learning, ANN, deep learning	11	Explore the evaluation and use of AI in breast imaging Explore the application of AI in breast imaging from the ethical, technical, and legal perspective
Purpose Specific Review					
48	[129]	Mammogram, ultrasound, SWE, SWV, and sonoelastography	CADx, SVM, CNN, LASSO, and ridge regression	4	Highlight the functions of ultrasomics and its capability in disease diagnostic (precision medicine) in different organs Highlight the limitations, challenges, and opportunities in ultrasomics application
49	[130]	Mammogram, ultrasound, and MRI	Deep learning and radiogenomics	13	Highlight the functions and integration process of radiogenomics Remark the strategies and relevant algorithms in such application
50	[131]	Mammogram, ultrasound, and tomosynthesis	Machine learning and deep learning	54	Highlight the functions and role of AI in early detection of breast cancer Highlight the readiness of AI in breast cancer imaging
51	[132]	EIT	PSO, ANN, GA, and other machine learning algorithms	44	Highlight the functions and roles of EIT and its application with AI
52	[133]	DOT	Deep learning	3	Highlight the roles, background, and relevant tools of AI in DOT
53	[134]	Ultrasound	Deep learning	2	Highlight the roles of AI in cancer, dermatology, cardiology, respiratory problems, neurodegenerative disorders, and gastroenterology
54	[135]	PET/CT and PET/MRI	Radiomics	16	Highlight the solutions and challenges of radiomics application in hybrid PET/CT and PET/MRI
55	[136]	Mammogram	CADe and CADx	28	Highlight the roles of CADe and CADx in mammography
56	[137]	Mammogram, ultrasound, DBT, and MRI	Deep learning, CADe, and CADx	3	Highlight the functions of AI applications in medical tasks and reasons for AI to be classified as a medical device Review the functions of CADe and its sub-elements Outline the FDA process in approving the CADe software
57	[138]	Ultrasound	CADe and CNN	0	Highlight the variation of ultrasound for dense breast imaging amongst average risk women
58	[139]	Thermography	SVM, ANN, BN, CADe, CNN, and GA	0	Highlight the roles of AI in thermography for early detection in breast cancer
59	[140]	Thermography	ANN	13	Highlight the roles of infrared thermography in breast imaging associated with clinical evidence that favor and unfavored such application Outline the historical timeline of infrared thermography
Systematic Review					
60	[141]	Mammogram, ultrasound, MRI, DBPET, DWI, PWI, CT, PET/CT, and PET/MRI	Radiomics, machine learning, and deep learning	5	Analyze and compare the conventional and advent breast imaging techniques Highlight the roles and future perspectives of advent breast imaging techniques in prediction of the response to NAC
61	[142]	Thermography	SVM, ANN, DNN, and RNN	81	Analyze and compare the studies in breast cancer detection using CADe and deep learning models
62	[143]	Mammogram, CT, and MRI	Machine learning, deep learning, and ANN,	1	Analyze the potential of AI in medical imaging Discuss the methodology, application, limitations, challenges, and future perspective in radiology
63	[144]	Mammogram, ultrasound, CT, and MRI	Deep CNN	2	Systematic review of current progress and state-of-the-art methods in intrafractional target motion management in breast cancer radiation therapy
64	[65]	Mammogram, DCE-MRI	Machine learning and deep learning	3	Systematic survey on 185 papers in cancer prediction and diagnostic using AI
65	[64]	Mammogram, ultrasound	CNN, ANN, DNN, MLP, SVM, DT, GA, KNN, NB, LR, LA, and GMM	12	Structured review on image processing, machine learning, and deep learning in breast imaging
Mixed Method Review					
66	[145]	Ultrasound and tomosynthesis	DNN, RCNN, faster RCNN, deep CNN, and ReLU	4	Commentary on current progress of DNN in computational radiology in the context of breast imaging and diagnostic
67	[146]	Mammogram, tomosynthesis, ultrasound, tomography, and MRI	Multilayered DNN	12	Review of novel approaches in breast imaging aided with AI
68	[147]	Mammogram and MRI	Radiomics	22	Review of various medical imaging modalities in the context of early detection Highlight the applications of various medical imaging modalities, the respective benefits, and the application of radiomics in such modalities
69	[148]	MRI	Machine learning and transfer learning	0	Systematic survey on application of machine learning in assessment of ALNM using MRI
70	[149]	Mammogram, ultrasound, and MRI	ANN, SNN, CNN, and CADe	41	Systematic survey of breast cancer classification in different breast imaging modalities aided by DNN approaches
Qualitative Review					
71	[150]	Mammogram, CT, and MRI	Machine learning	51	Qualitative survey of the impact of AI on radiology

* Data retrieved from the Scopus database as of 17 June 2022. AI: artificial intelligence; CNN: Convolutional Neural Networks; ANN: Artificial Neural Network; RBFN: Radial Basis Function Network; KNN: K-Nearest Neighbors; PNN: Probability Neural Network; SVM: Support Vector Machine; C-DCNN: DeConvolutional Neural Networks; CT: Computer tomography; MRI: magnetic resonance imaging; FDG: fluorodeoxyglucose; PET: Positron Emission Tomography; CADe: computer-aided detection; NLP: natural language processing; 3D: three dimensional; DBCT: Dedicated breast computed tomography; DWI: diffusion-weighted imaging, NIR: Near-Infrared; SPECT: Single-photon emission computed tomography; CADx: computer-aided diagnosis; DCE-MRI: dynamic contrast-enhanced magnetic resonance imaging; DCIS: ductal carcinoma in situ; GANs: generative adversarial networks; DBT: digital breast tomosynthesis; CEDM: Contrast-enhanced digital mammography; SNN: Shallow Neural Network; SDAE: Stacked Denoising Autoencoder; DBN: Deep Belief Network; DNN: deep neural networks; IoT: Internet of things; MBI: molecular breast imaging; PACS: picture archiving and communications system; SWE: shear wave elastography; SWV: shear wave viscosity; LASSO: least absolute shrinkage and selection operator; MRE: Magnetic resonance elastography; EIT: Electrical impedance tomography; PSO: particle swarm optimization; GA: genetic algorithm; DOT: Diffuse optical tomography; BN: Bayesian network; CE-MRI: contrast-enhanced magnetic resonance imaging; NAC: Neoadjuvant chemotherapy; DBPET: Dedicated breast positron emission tomography; PWI: perfusion weighted imaging; RNN: recurrent neural network; MLP: Multi-layer Perceptron; DT: decision tree; NB: naïve bayes; LR: Logistic Regression; LA: Linear discriminant analysis; GMM: Gaussian Mixture Modelling; RCNN: Region-Based Convolutional Neural Network; ReLU: rectified linear unit; ALNM: axillary lymph node metastasis.

**Table A2:** AMSTAR rating for each included review work (systematic review).

No.	References	Items										
		1	2	3	4	5	6	7	8	9	10	11
Systematic Review												
1	[141]	−	−	−	−	−	−	−	+	∅	−	+
2	[142]	−	−	−	−	−	−	−	+	∅	−	+
3	[143]	−	+	+	−	+	+	+	+	+	−	+
4	[144]	−	+	+	−	+	+	−	+	−	−	+
5	[65]	+	+	+	−	+	+	+	+	+	−	+
6	[64]	+	+	+	+	+	−	+	+	+	+	+
Analysis												
	+	2	4	4	1	4	3	3	6	3	1	6
	−	4	2	2	5	2	3	3	0	1	5	0
	⊗	0	0	0	0	0	0	0	0	0	0	0
	∅	0	0	0	0	0	0	0	0	2	0	0
	Total	6										

+: Yes; −: No; ⊗: Can’t answer; ∅: Not applicable.

**Table A3:** SANRA rating for each included review work (non-systematic review).

No.	References	Items					
		1	2	3	4	5	6
Traditional Review							
1	[84]	2	2	0	2	2	1
2	[85]	2	2	0	2	2	1
3	[86]	1	1	0	2	1	1
4	[87]	2	1	0	2	2	1
5	[88]	1	2	0	2	1	2
6	[89]	1	1	2	1	1	1
7	[90]	2	2	0	2	1	1
8	[91]	2	1	0	2	2	2
9	[92]	2	2	0	2	2	2
10	[93]	2	1	1	2	2	2
11	[94]	2	2	0	2	1	0
12	[9]	2	1	0	2	2	2
13	[95]	1	1	0	1	2	2
14	[96]	1	2	2	2	2	2
15	[97]	1	0	0	2	2	0
16	[98]	1	1	2	1	0	0
17	[99]	1	2	0	1	1	1
18	[100]	1	1	0	2	2	1
19	[101]	1	1	0	1	1	0
20	[102]	2	1	2	2	2	2
21	[103]	2	2	0	1	2	2
22	[104]	2	1	0	2	1	0
23	[105]	1	1	0	2	1	0
24	[106]	2	1	1	2	2	2
25	[70]	2	2	0	2	2	1
26	[107]	2	2	0	2	1	0
27	[108]	1	0	0	1	1	0
28	[109]	2	2	1	1	2	2
29	[110]	2	1	2	2	2	1
30	[111]	1	1	2	2	2	0
31	[112]	1	1	0	2	2	2
32	[113]	2	2	0	2	2	0
33	[114]	2	2	0	2	2	2
34	[115]	2	1	0	2	2	0
35	[116]	2	1	2	2	2	2
36	[117]	1	1	0	2	2	0
37	[118]	2	2	0	2	2	2
38	[119]	2	2	0	2	2	0
39	[120]	1	2	0	2	2	2
40	[121]	2	0	0	1	1	0
41	[122]	1	1	1	2	2	2
42	[123]	2	1	2	2	2	2
43	[124]	2	2	0	2	2	2
44	[125]	2	2	0	1	1	0
45	[126]	2	2	0	2	2	1
46	[127]	1	1	0	1	1	0
47	[128]	1	1	0	1	1	0
Purpose Specific Review							
48	[129]	1	1	0	1	1	1
49	[130]	2	1	0	2	2	1
50	[131]	2	2	2	1	2	2
51	[132]	1	1	0	2	2	1
52	[133]	1	2	0	2	2	1
53	[134]	1	1	0	2	1	1
54	[135]	2	2	0	2	2	2
55	[136]	2	1	0	2	2	2
56	[137]	2	2	0	1	1	0
57	[138]	1	2	0	2	2	2
58	[139]	2	1	0	2	1	2
59	[140]	1	2	0	1	2	2
Mixed Method Review							
60	[145]	2	1	0	1	1	0
61	[146]	1	1	0	2	2	2
62	[147]	1	1	0	2	2	1
63	[148]	2	2	2	1	2	2
64	[149]	2	2	2	2	2	2
Qualitative Review							
65	[150]	2	1	0	0	2	2
Analysis							
	Score 0	0	3	50	1	1	19
	Score 1	27	34	4	18	20	17
	Score 2	38	28	11	46	44	29
	Total	65					

## References

[1] Faguet G.B. (2015). A brief history of cancer: Age-old milestones underlying our current knowledge database. Int. J. Cancer.

[2] US National Library of Medicine. An ancient Medical Treasure at your fingertips. *NLM Tech Bull*
**2010**. https://www.nlm.nih.gov/pubs/techbull/ma10/ma10_hmd_reprint_papyrus.html.

[3] Sung H., Ferlay J., Siegel R.L., Laversanne M., Soerjomataram I., Jemal A., Bray F. (2021). Global Cancer Statistics 2020: GLOBOCAN Estimates of Incidence and Mortality Worldwide for 36 Cancers in 185 Countries. CA Cancer J. Clin..

[4] Chu J., Zhou C., Guo X., Sun J., Xue F., Zhang J., Lu Z., Fu Z., Xu A. (2017). Female Breast Cancer Mortality Clusters in Shandong Province, China: A Spatial Analysis. Sci. Rep..

[5] Akin O., Brennan S., Dershaw D.D., Ginsberg M.S., Gollub M.J., Schöder H., Panicek D., Hricak H. (2012). Advances in oncologic imaging. CA Cancer J. Clin..

[6] Evans W.P. (2012). Breast cancer screening: Successes and challenges. CA Cancer J. Clin..

[7] Duffy S.W., Tabár L., Yen A.M., Dean P.B., Smith R.A., Jonsson H., Törnberg S., Chen S.L.-S., Chiu S.Y., Fann J.C. (2020). Mammography screening reduces rates of advanced and fatal breast cancers: Results in 549,091 women. Cancer.

[8] Napel S., Mu W., Jardim-Perassi B.V., Aerts H.J.W.L., Gillies R.J. (2018). Quantitative imaging of cancer in the postgenomic era: Radio(geno)mics, deep learning, and habitats. Cancer.

[9] Bi W.L., Hosny A., Schabath M.B., Giger M.L., Birkbak N., Mehrtash A., Allison T., Arnaout O., Abbosh C., Dunn I.F. (2019). Artificial intelligence in cancer imaging: Clinical challenges and applications. CA Cancer J. Clin..

[10] Yu J., Deng Y., Liu T., Zhou J., Jia X., Xiao T., Zhou S., Li J., Guo Y., Wang Y. (2020). Lymph node metastasis prediction of papillary thyroid carcinoma based on transfer learning radiomics. Nat. Commun..

[11] Wang Y., Herrington D.M. (2021). Machine intelligence enabled radiomics. Nat. Mach. Intell..

[12] Roy S., Whitehead T.D., Quirk J.D., Salter A., Ademuyiwa F.O., Li S., An H., Shoghi K.I. (2020). Optimal co-clinical radiomics: Sensitivity of radiomic features to tumour volume, image noise and resolution in co-clinical T1-weighted and T2-weighted magnetic resonance imaging. Ebiomedicine.

[13] Clarke L.P., Nordstrom R.J., Zhang H., Tandon P., Zhang Y., Redmond G., Farahani K., Kelloff G., Henderson L., Shankar L. (2014). The Quantitative Imaging Network: NCI’s Historical Perspective and Planned Goals. Transl. Oncol..

[14] Nordstrom R.J. (2016). The Quantitative Imaging Network in Precision Medicine. Tomography.

[15] Van Timmeren J.E., Cester D., Tanadini-Lang S., Alkadhi H., Baessler B. (2020). Radiomics in medical imaging-”how-to” guide and critical reflection. Insights Imaging.

[16] Rogers W., Seetha S.T., Refaee T.A.G., Lieverse R.I.Y., Granzier R.W.Y., Ibrahim A., Keek S.A., Sanduleanu S., Primakov S.P., Beuque M.P.L. (2020). Radiomics: From qualitative to quantitative imaging. Br. J. Radiol..

[17] Gillies R.J., Kinahan P.E., Hricak H. (2016). Radiomics: Images Are More than Pictures, They Are Data. Radiology.

[18] Aerts H.J.W.L. (2016). The Potential of Radiomic-Based Phenotyping in Precision Medicine. JAMA Oncol..

[19] Roy S., Whitehead T.D., Li S., Ademuyiwa F.O., Wahl R.L., Dehdashti F., Shoghi K.I. (2022). Co-clinical FDG-PET radiomic signature in predicting response to neoadjuvant chemotherapy in triple-negative breast cancer. Eur. J. Nucl. Med..

[20] Mnih V., Kavukcuoglu K., Silver D., Rusu A.A., Veness J., Bellemare M.G., Graves A., Riedmiller M., Fidjeland A.K., Ostrovski G. (2015). Human-level control through deep reinforcement learning. Nature.

[21] LeCun Y., Bengio Y., Hinton G. (2015). Deep learning. Nature.

[22] Moravčík M., Schmid M., Burch N., Lisý V., Morrill D., Bard N., Davis T., Waugh K., Johanson M., Bowling M. (2017). DeepStack: Expert-level artificial intelligence in heads-up no-limit poker. Science.

[23] Xiong W., Droppo J., Huang X., Seide F., Seltzer M.L., Stolcke A., Yu D., Zweig G. (2017). Toward Human Parity in Conversational Speech Recognition. IEEE/ACM Trans. Audio Speech Lang. Process..

[24] Pendleton S.D., Andersen H., Du X., Shen X., Meghjani M., Eng Y.H., Rus D., Ang M. (2017). Perception, Planning, Control, and Coordination for Autonomous Vehicles. Machines.

[25] Hosny A., Parmar C., Quackenbush J., Schwartz L.H., Aerts H.J.W.L. (2018). Artificial intelligence in radiology. Nat. Rev. Cancer.

[26] Sutton A., Clowes M., Preston L., Booth A. (2019). Meeting the review family: Exploring review types and associated information retrieval requirements. Health Inf. Libr. J..

[27] Slim K., Marquillier T. (2022). Umbrella reviews: A new tool to synthesize scientific evidence in surgery. J. Visc. Surg..

[28] Chen C., Song M. (2019). Visualizing a field of research: A methodology of systematic scientometric reviews. PLoS ONE.

[29] Kalaf J.M. (2014). Mammography: A history of success and scientific enthusiasm. Radiol. Bras..

[30] Trivedi U., Omofoye T.S., Marquez C., Sullivan C.R., Benson D.M., Whitman G.J. (2022). Mobile Mammography Services and Underserved Women. Diagnostics.

[31] Rao V.M., Levin D.C., Parker L., Cavanaugh B., Frangos A.J., Sunshine J.H. (2010). How Widely Is Computer-Aided Detection Used in Screening and Diagnostic Mammography?. J. Am. Coll. Radiol..

[32] Britt K.L., Cuzick J., Phillips K.-A. (2020). Key steps for effective breast cancer prevention. Nat. Rev. Cancer.

[33] Loibl S., Poortmans P., Morrow M., Denkert C., Curigliano G. (2021). Breast cancer. Lancet.

[34] Iranmakani S., Mortezazadeh T., Sajadian F., Ghaziani M.F., Ghafari A., Khezerloo D., Musa A.E. (2020). A review of various modalities in breast imaging: Technical aspects and clinical outcomes. Egypt. J. Radiol. Nucl. Med..

[35] Friedewald S.M. (2018). Breast Cancer Screening: The Debate that Never Ends. Optim. Breast Cancer Manag..

[36] Scheel J.R., Lee J.M., Sprague B.L., Lee C.I., Lehman C.D. (2015). Screening ultrasound as an adjunct to mammography in women with mammographically dense breasts. Am. J. Obstet. Gynecol..

[37] Ohuchi N., Suzuki A., Sobue T., Kawai M., Yamamoto S., Zheng Y.-F., Shiono Y.N., Saito H., Kuriyama S., Tohno E. (2016). Sensitivity and specificity of mammography and adjunctive ultrasonography to screen for breast cancer in the Japan Strategic Anti-cancer Randomized Trial (J-START): A randomised controlled trial. Lancet.

[38] Harada-Shoji N., Suzuki A., Ishida T., Zheng Y.-F., Narikawa-Shiono Y., Sato-Tadano A., Ohta R., Ohuchi N. (2021). Evaluation of Adjunctive Ultrasonography for Breast Cancer Detection Among Women Aged 40–49 Years With Varying Breast Density Undergoing Screening Mammography. JAMA Netw. Open.

[39] Kaul V., Enslin S., Gross S.A. (2020). History of artificial intelligence in medicine. Gastrointest. Endosc..

[40] Hamamoto R., Suvarna K., Yamada M., Kobayashi K., Shinkai N., Miyake M., Takahashi M., Jinnai S., Shimoyama R., Sakai A. (2020). Application of Artificial Intelligence Technology in Oncology: Towards the Establishment of Precision Medicine. Cancers.

[41] Luchini C., Pea A., Scarpa A. (2022). Artificial intelligence in oncology: Current applications and future perspectives. Br. J. Cancer.

[42] Teare P., Fishman M., Benzaquen O., Toledano E., Elnekave E. (2017). Malignancy Detection on Mammography Using Dual Deep Convolutional Neural Networks and Genetically Discovered False Color Input Enhancement. J. Digit. Imaging.

[43] Shenbagavalli P., Thangarajan R. (2018). Aiding the Digital Mammogram for Detecting the Breast Cancer Using Shearlet Transform and Neural Network. Asian Pac. J. Cancer Prev..

[44] Becker A., Marcon M., Ghafoor S., Wurnig M.C., Frauenfelder T., Boss A. (2017). Deep Learning in Mammography. Investig. Radiol..

[45] Oren O., Gersh B.J., Bhatt D.L. (2020). Artificial intelligence in medical imaging: Switching from radiographic pathological data to clinically meaningful endpoints. Lancet Digit. Health.

[46] Heuvel T.V.D., van der Eerden A., Manniesing R., Ghafoorian M., Tan T., Andriessen T., Vyvere T.V., Hauwe L.V.D., Romeny B.T.H., Goraj B. (2016). Automated detection of cerebral microbleeds in patients with traumatic brain injury. NeuroImage Clin..

[47] Greene F.L., Sobin L.H. (2008). The Staging of Cancer: A Retrospective and Prospective Appraisal. CA Cancer J. Clin..

[48] Santos M., Gomes C., Marcos R., Santos A., De Matos A., Lopes C., Dias-Pereira P. (2015). Value of the Nottingham Histological Grading Parameters and Nottingham Prognostic Index in Canine Mammary Carcinoma. Anticancer Res..

[49] Jaffe C.C. (2006). Measures of Response: RECIST, WHO, and New Alternatives. J. Clin. Oncol..

[50] Guenther L.M., Rowe R.G., Acharya P.T., Swenson D.W., Meyer S.C., Clinton C.M., Guo D., Sridharan M., London W.B., Grier H.E. (2018). Response Evaluation Criteria in Solid Tumors (RECIST) following neoadjuvant chemotherapy in osteosarcoma. Pediatr. Blood Cancer.

[51] Laney D. (2001). 3D data management: Controlling data volume, velocity, and variety. META Group Res. Note.

[52] Dash S., Shakyawar S.K., Sharma M., Kaushik S. (2019). Big data in healthcare: Management, analysis and future prospects. J. Big Data.

[53] Page M.J., McKenzie J.E., Bossuyt P.M., Boutron I., Hoffmann T.C., Mulrow C.D., Shamseer L., Tetzlaff J.M., Akl E.A., Brennan S.E. (2021). The PRISMA 2020 statement: An updated guideline for reporting systematic reviews. Int. J. Surg..

[54] Shea B.J., Grimshaw J.M., A Wells G., Boers M., Andersson N., Hamel C., Porter A.C., Tugwell P., Moher D., Bouter L.M. (2007). Development of AMSTAR: A measurement tool to assess the methodological quality of systematic reviews. BMC Med. Res. Methodol..

[55] Baethge C., Goldbeck-Wood S., Mertens S. (2019). SANRA—A scale for the quality assessment of narrative review articles. Res. Integr. Peer Rev..

[56] Perianes-Rodriguez A., Waltman L., van Eck N.J. (2016). Constructing bibliometric networks: A comparison between full and fractional counting. J. Inf..

[57] Larivière V., Haustein S., Mongeon P. (2015). The Oligopoly of Academic Publishers in the Digital Era. PLoS ONE.

[58] Association, I.P. The Global Publishing Industry in 2018. https://www.wipo.int/edocs/pubdocs/en/wipo_pub_1064_2019.pdf.

[59] Tan X.J., Cheor W.L., Yeo K.S., Leow W.Z. (2022). Expert systems in oil palm precision agriculture: A decade systematic review. J. King Saud Univ. -Comput. Inf. Sci..

[60] Momeni F., Mayr P., Fraser N., Peters I. (2021). What happens when a journal converts to open access? A bibliometric analysis. Scientometrics.

[61] Gaston T.E., Ounsworth F., Senders T., Ritchie S., Jones E. (2020). Factors affecting journal submission numbers: Impact factor and peer review reputation. Learn. Publ..

[62] Subramanyam K. (1983). Bibliometric studies of research collaboration: A review. J. Inf. Sci..

[63] Egghe L., Rousseau R. (2002). Co-citation, bibliographic coupling and a characterization of lattice citation networks. Scientometrics.

[64] Zerouaoui H., Idri A. (2021). Reviewing Machine Learning and Image Processing Based Decision-Making Systems for Breast Cancer Imaging. J. Med. Syst..

[65] Kumar Y., Gupta S., Singla R., Hu Y.-C. (2022). A Systematic Review of Artificial Intelligence Techniques in Cancer Prediction and Diagnosis. Arch. Comput. Methods Eng..

[66] Pieper D., Lorenz R.C., Rombey T., Jacobs A., Rissling O., Freitag S., Matthias K. (2021). Authors should clearly report how they derived the overall rating when applying AMSTAR 2—A cross-sectional study. J. Clin. Epidemiol..

[67] Wang S., Li C., Wang R., Liu Z., Wang M., Tan H., Wu Y., Liu X., Sun H., Yang R. (2021). Annotation-efficient deep learning for automatic medical image segmentation. Nat. Commun..

[68] Aganj I., Harisinghani M.G., Weissleder R., Fischl B. (2018). Unsupervised Medical Image Segmentation Based on the Local Center of Mass. Sci. Rep..

[69] Purushotham S., Meng C., Che Z., Liu Y. (2018). Benchmarking deep learning models on large healthcare datasets. J. Biomed. Inform..

[70] Hickman S.E., Baxter G.C., Gilbert F.J. (2021). Adoption of artificial intelligence in breast imaging: Evaluation, ethical constraints and limitations. Br. J. Cancer.

[71] Van Ginneken B., Schaefer-Prokop C.M., Prokop M. (2011). Computer-aided Diagnosis: How to Move from the Laboratory to the Clinic. Radiology.

[72] Gilad-Bachrach R., Dowlin N., Laine K., Lauter K., Naehrig M., Wernsing J. (2016). CryptoNets: Applying Neural Networks to Encrypted Data with High Throughput and Accuracy. Microsoft Res. Technol. Rep..

[73] George A., Walsh T. (2022). Artificial intelligence is breaking patent law. Nature.

[74] Serafimova S. (2020). Whose morality? Which rationality? Challenging artificial intelligence as a remedy for the lack of moral enhancement. Humanit. Soc. Sci. Commun..

[75] Köbis N., Bonnefon J.-F., Rahwan I. (2021). Bad machines corrupt good morals. Nat. Hum. Behav..

[76] Gordon R. (2022). Artificial intelligence predicts patients’ race from their medical images. MIT News.

[77] Tsamados A., Aggarwal N., Cowls J., Morley J., Roberts H., Taddeo M., Floridi L. (2022). The ethics of algorithms: Key problems and solutions. AI Soc..

[78] Barrett S.R.H., Speth R., Eastham S., Dedoussi I.C., Ashok A., Malina R., Keith D.W. (2015). Impact of the Volkswagen emissions control defeat device on US public health. Environ. Res. Lett..

[79] (2022). Challenges in digital medicine applications in under-resourced settings. Nat. Commun..

[80] Vourgidis I., Mafuma S.J., Wilson P., Carter J., Cosma G. (2018). Medical Expert Systems—A Study of Trust and Acceptance by Healthcare Stakeholders.

[81] Feng J., Phillips R.V., Malenica I., Bishara A., Hubbard A.E., Celi L.A., Pirracchio R. (2022). Clinical artificial intelligence quality improvement: Towards continual monitoring and updating of AI algorithms in healthcare. Npj Digit. Med..

[82] Wilson N. (2019). On the Road to Convergence Research. BioScience.

[83] Petersen A.M., Ahmed M.E., Pavlidis I. (2021). Grand challenges and emergent modes of convergence science. Humanit. Soc. Sci. Commun..

[84] Roslidar R., Rahman A., Muharar R., Syahputra M.R., Arnia F., Syukri M., Pradhan B., Munadi K. (2020). A Review on Recent Progress in Thermal Imaging and Deep Learning Approaches for Breast Cancer Detection. IEEE Access.

[85] Al Husaini M.A.S., Habaebi M.H., Hameed S.A., Islam R., Gunawan T.S. (2020). A Systematic Review of Breast Cancer Detection Using Thermography and Neural Networks. IEEE Access.

[86] Panayides A.S., Amini A., Filipovic N.D., Sharma A., Tsaftaris S.A., Young A.A., Foran D.J., Do N.V., Golemati S., Kurc T. (2020). AI in Medical Imaging Informatics: Current Challenges and Future Directions. IEEE J. Biomed. Health Inform..

[87] Lei Y.-M., Yin M., Yu M.-H., Yu J., Zeng S.-E., Lv W.-Z., Li J., Ye H.-R., Cui X.-W., Dietrich C.F. (2021). Artificial Intelligence in Medical Imaging of the Breast. Front. Oncol..

[88] Arieno A., Chan A., Destounis S.V. (2019). A Review of the Role of Augmented Intelligence in Breast Imaging: From Automated Breast Density Assessment to Risk Stratification. Am. J. Roentgenol..

[89] Ellen B.M. (2019). Artificial Intelligence in Breast Imaging: Potentials and Limitations. Am. J. Roentgenol..

[90] Cui C., Chou S.-H.S., Brattain L., Lehman C.D., Samir A.E. (2019). Data Engineering for Machine Learning in Women’s Imaging and Beyond. Am. J. Roentgenol..

[91] Zuluaga-Gomez J., Zerhouni N., Al Masry Z., Devalland C., Varnier C. (2019). A survey of breast cancer screening techniques: Thermography and electrical impedance tomography. J. Med. Eng. Technol..

[92] Pal U.M., Saxena M., Vishnu G.K.A., Parsana D., Sarvani B.S.R., Varma M., Jayachandra M., Kurpad V., Baruah D., Gogoi G. (2020). Optical spectroscopy-based imaging techniques for the diagnosis of breast cancer: A novel approach. Appl. Spectrosc. Rev..

[93] Aruleba K., Obaido G., Ogbuokiri B., Fadaka A., Klein A., Adekiya T., Aruleba R. (2020). Applications of Computational Methods in Biomedical Breast Cancer Imaging Diagnostics: A Review. J. Imaging.

[94] Bashir U., Mallia A., Stirling J., Joemon J., MacKewn J., Charles-Edwards G., Goh V., Cook G.J. (2015). PET/MRI in Oncological Imaging: State of the Art. Diagnostics.

[95] Sheth D., Giger M.L. (2020). Artificial intelligence in the interpretation of breast cancer on MRI. J. Magn. Reson. Imaging.

[96] Meyer-Bäse A., Morra L., Meyer-Bäse U., Pinker K. (2020). Current Status and Future Perspectives of Artificial Intelligence in Magnetic Resonance Breast Imaging. Contrast Media Mol. Imaging.

[97] Greenwood H.I., Wilmes L.J., Kelil T., Joe B.N. (2019). Role of Breast MRI in the Evaluation and Detection of DCIS: Opportunities and Challenges. J. Magn. Reson. Imaging.

[98] Goyal S. (2021). An Overview of Current Trends, Techniques, Prospects, and Pitfalls of Artificial Intelligence in Breast Imaging. Rep. Med. Imaging.

[99] Bahl M. (2020). Artificial Intelligence: A Primer for Breast Imaging Radiologists. J. Breast Imaging.

[100] Geras K.J., Mann R.M., Moy L. (2019). Artificial Intelligence for Mammography and Digital Breast Tomosynthesis: Current Concepts and Future Perspectives. Radiology.

[101] Kim J., Kim H.J., Kim C., Kim W.H. (2021). Artificial intelligence in breast ultrasonography. Ultrasonography.

[102] Chan H.-P., Samala R.K., Hadjiiski L.M. (2020). CAD and AI for breast cancer—Recent development and challenges. Br. J. Radiol..

[103] Jochelson M.S., Lobbes M.B.I. (2021). Contrast-enhanced Mammography: State of the Art. Radiology.

[104] de Figueiredo G.N., Ingrisch M., Fallenberg E.M. (2019). Digital Analysis in Breast Imaging. Breast Care.

[105] Dietzel M., Clauser P., Kapetas P., Schulz-Wendtland R., Baltzer P.A.T. (2021). Images Are Data: A Breast Imaging Perspective on a Contemporary Paradigm. Rofo.

[106] Yi J., Kang H.K., Kwon J.-H., Kim K.-S., Park M.H., Seong Y.K., Kim D.W., Ahn B., Ha K., Lee J. (2021). Technology trends and applications of deep learning in ultrasonography: Image quality enhancement, diagnostic support, and improving workflow efficiency. Ultrasonography.

[107] Gandomkar Z., Mello-Thoms C. (2019). Visual search in breast imaging. Br. J. Radiol..

[108] Fujita H. (2020). AI-based computer-aided diagnosis (AI-CAD): The latest review to read first. Radiol. Phys. Technol..

[109] Gastounioti A., Desai S., Ahluwalia V.S., Conant E.F., Kontos D. (2022). Artificial intelligence in mammographic phenotyping of breast cancer risk: A narrative review. Breast Cancer Res..

[110] Decuyper M., Maebe J., Van Holen R., Vandenberghe S. (2021). Artificial intelligence with deep learning in nuclear medicine and radiology. EJNMMI Phys..

[111] Pesapane F., Codari M., Sardanelli F. (2018). Artificial intelligence in medical imaging: Threat or opportunity? Radiologists again at the forefront of innovation in medicine. Eur. Radiol. Exp..

[112] Ou W.C., Polat D., Dogan B.E. (2021). Deep learning in breast radiology: Current progress and future directions. Eur. Radiol..

[113] Bitencourt A., Naranjo I.D., Gullo R.L., Saccarelli C.R., Pinker K. (2021). AI-enhanced breast imaging: Where are we and where are we heading?. Eur. J. Radiol..

[114] Shah S.M., Khan R.A., Arif S., Sajid U. (2022). Artificial intelligence for breast cancer analysis: Trends & directions. Comput. Biol. Med..

[115] Le E., Wang Y., Huang Y., Hickman S., Gilbert F. (2019). Artificial intelligence in breast imaging. Clin. Radiol..

[116] Huang S., Yang J., Fong S., Zhao Q. (2019). Artificial intelligence in cancer diagnosis and prognosis: Opportunities and challenges. Cancer Lett..

[117] Shen Y.-T., Chen L., Yue W.-W., Xu H.-X. (2021). Artificial intelligence in ultrasound. Eur. J. Radiol..

[118] Barba D., León-Sosa A., Lugo P., Suquillo D., Torres F., Surre F., Trojman L., Caicedo A. (2021). Breast cancer, screening and diagnostic tools: All you need to know. Crit. Rev. Oncol..

[119] Cheung H., Rubin D. (2021). Challenges and opportunities for artificial intelligence in oncological imaging. Clin. Radiol..

[120] Shiraishi J., Li Q., Appelbaum D., Doi K. (2011). Computer-Aided Diagnosis and Artificial Intelligence in Clinical Imaging. Semin. Nucl. Med..

[121] Chen D.-R., Hsiao Y.-H. (2008). Computer-aided Diagnosis in Breast Ultrasound. J. Med. Ultrasound.

[122] Houssein E.H., Emam M.M., Ali A.A., Suganthan P.N. (2021). Deep and machine learning techniques for medical imaging-based breast cancer: A comprehensive review. Expert Syst. Appl..

[123] Siviengphanom S., Gandomkar Z., Lewis S.J., Brennan P.C. (2021). Mammography-based Radiomics in Breast Cancer: A Scoping Review of Current Knowledge and Future Needs. Acad. Radiol..

[124] Yin X.-X., Hadjiloucas S., Zhang Y., Tian Z. (2022). MRI radiogenomics for intelligent diagnosis of breast tumors and accurate prediction of neoadjuvant chemotherapy responses-a review. Comput. Methods Prog. Biomed..

[125] Ghieh D., Saade C., Najem E., El Zeghondi R., Rawashdeh M., Berjawi G. (2021). Staying abreast of imaging—urrent status of breast cancer detection in high density breast. Radiography.

[126] Gonzalez-Hernandez J.-L., Recinella A.N., Kandlikar S.G., Dabydeen D., Medeiros L., Phatak P. (2019). Technology, application and potential of dynamic breast thermography for the detection of breast cancer. Int. J. Heat Mass Transf..

[127] Fazal M.I., Patel M.E., Tye J., Gupta Y. (2018). The past, present and future role of artificial intelligence in imaging. Eur. J. Radiol..

[128] Mutasa S., Sun S., Ha R. (2021). Understanding artificial intelligence based radiology studies: CNN architecture. Clin. Imaging.

[129] Yin R., Jiang M., Lv W.-Z., Jiang F., Li J., Hu B., Cui X.-W., Dietrich C.F. (2020). Study Processes and Applications of Ultrasomics in Precision Medicine. Front. Oncol..

[130] Shui L., Ren H., Yang X., Li J., Chen Z., Yi C., Zhu H., Shui P. (2021). The Era of Radiogenomics in Precision Medicine: An Emerging Approach to Support Diagnosis, Treatment Decisions, and Prognostication in Oncology. Front. Oncol..

[131] Houssami N., Kirkpatrick-Jones G., Noguchi N., Lee C.I. (2019). Artificial Intelligence (AI) for the early detection of breast cancer: A scoping review to assess AI’s potential in breast screening practice. Expert Rev. Med. Dev..

[132] Khan T.A., Ling S.H. (2019). Review on Electrical Impedance Tomography: Artificial Intelligence Methods and its Applications. Algorithms.

[133] Balasubramaniam G.M., Wiesel B., Biton N., Kumar R., Kupferman J., Arnon S. (2022). Tutorial on the Use of Deep Learning in Diffuse Optical Tomography. Electronics.

[134] Olveres J., González G., Torres F., Moreno-Tagle J.C., Carbajal-Degante E., Valencia-Rodríguez A., Méndez-Sánchez N., Escalante-Ramírez B. (2021). What is new in computer vision and artificial intelligence in medical image analysis applications. Quant. Imaging Med. Surg..

[135] Castiglioni I., Gallivanone F., Soda P., Avanzo M., Stancanello J., Aiello M., Interlenghi M., Salvatore M. (2019). AI-based applications in hybrid imaging: How to build smart and truly multi-parametric decision models for radiomics. Eur. J. Nucl. Med..

[136] Bazzocchi M., Mazzarella F., Del Frate C., Girometti F., Zuiani C. (2007). CAD systems for mammography: A real opportunity? A review of the literature. Radiol. Med..

[137] Retson T.A., Eghtedari M. (2020). Computer-Aided Detection/Diagnosis in Breast Imaging: A Focus on the Evolving FDA Regulations for Using Software as a Medical Device. Curr. Radiol. Rep..

[138] Spear G.G., Mendelson E.B. (2020). Automated breast ultrasound: Supplemental screening for average-risk women with dense breasts. Clin. Imaging.

[139] Mashekova A., Zhao Y., Ng E.Y., Zarikas V., Fok S.C., Mukhmetov O. (2022). Early detection of the breast cancer using infrared technology—A comprehensive review. Therm. Sci. Eng. Prog..

[140] Lozano A., Hassanipour F. (2019). Infrared imaging for breast cancer detection: An objective review of foundational studies and its proper role in breast cancer screening. Infrared Phys. Technol..

[141] Romeo V., Accardo G., Perillo T., Basso L., Garbino N., Nicolai E., Maurea S., Salvatore M. (2021). Assessment and Prediction of Response to Neoadjuvant Chemotherapy in Breast Cancer: A Comparison of Imaging Modalities and Future Perspectives. Cancers.

[142] Mambou S.J., Maresova P., Krejcar O., Selamat A., Kuca K. (2018). Breast Cancer Detection Using Infrared Thermal Imaging and a Deep Learning Model. Sensors.

[143] Hameed B.Z., Prerepa G., Patil V., Shekhar P., Raza S.Z., Karimi H., Paul R., Naik N., Modi S., Vigneswaran G. (2021). Engineering and clinical use of artificial intelligence (AI) with machine learning and data science advancements: Radiology leading the way for future. Ther. Adv. Urol..

[144] Piruzan E., Vosoughi N., Mahdavi S.R., Khalafi L., Mahani H. (2021). Target motion management in breast cancer radiation therapy. Radiol. Oncol..

[145] Tran W.T., Sadeghi-Naini A., Lu F.-I., Gandhi S., Meti N., Brackstone M., Rakovitch E., Curpen B. (2020). Computational Radiology in Breast Cancer Screening and Diagnosis Using Artificial Intelligence. Can. Assoc. Radiol. J..

[146] Mann R.M., Hooley R., Barr R.G., Moy L. (2020). Novel Approaches to Screening for Breast Cancer. Radiology.

[147] Gillies R.J., Schabath M.B. (2020). Radiomics Improves Cancer Screening and Early Detection. Cancer Epidemiol. Biomark. Prev..

[148] Chen C., Qin Y., Chen H., Zhu D., Gao F., Zhou X. (2021). A meta-analysis of the diagnostic performance of machine learning-based MRI in the prediction of axillary lymph node metastasis in breast cancer patients. Insights Imaging.

[149] Murtaza G., Shuib L., Wahab A.W.A., Mujtaba G., Nweke H.F., Al-Garadi M.A., Zulfiqar F., Raza G., Azmi N.A. (2020). Deep learning-based breast cancer classification through medical imaging modalities: State of the art and research challenges. Artif. Intell. Rev..

[150] European Society of Radiology (ESR) (2019). Impact of artificial intelligence on radiology: A EuroAIM survey among members of the European Society of Radiology. Insights Imaging.

